# Cannabinoid receptor CNR1 expression and DNA methylation in human prefrontal cortex, hippocampus and caudate in brain development and schizophrenia

**DOI:** 10.1038/s41398-020-0832-8

**Published:** 2020-05-19

**Authors:** Ran Tao, Chao Li, Andrew E. Jaffe, Joo Heon Shin, Amy Deep-Soboslay, Rae’e Yamin, Daniel R. Weinberger, Thomas M. Hyde, Joel E. Kleinman

**Affiliations:** 1 The Lieber Institute for Brain Development, Johns Hopkins University Medical Campus, Baltimore, MD USA; 2 Department of Mental Health, Johns Hopkins Bloomberg School of Public Health, Baltimore, MD USA; 3 Department of Psychiatry and Behavior Sciences, Johns Hopkins School of Medicine, Baltimore, MD USA; 4 Department of Neurology, Johns Hopkins University School of Medicine, Baltimore, MD USA; 5 Department of Neuroscience, Johns Hopkins University School of Medicine, Baltimore, MD USA; 6 McKusick Nathans Institute of Genetic Medicine, Johns Hopkins University School of Medicine, Baltimore, MD USA

**Keywords:** Molecular neuroscience, Schizophrenia

## Abstract

Beyond being one the most widely used psychoactive drugs in the world, cannabis has been identified as an environmental risk factor for psychosis. Though the relationship between cannabis use and psychiatric disorders remains controversial, consistent association between early adolescent cannabis use and the subsequent risk of psychosis suggested adolescence may be a particularly vulnerable period. Previous findings on gene by environment interactions indicated that cannabis use may only increase the risk for psychosis in the subjects who have a specific genetic vulnerability. The type 1 cannabinoid receptor (CB1), encoded by the *CNR1* gene, is a key component of the endocannabinoid system. As the primary endocannabinoid receptor in the brain, CB1 is the main molecular target of the endocannabinoid ligand, as well as tetrahydrocannabinol (THC), the principal psychoactive ingredient of cannabis. In this study, we have examined mRNA expression and DNA methylation of *CNR1* in human prefrontal cortex (PFC), hippocampus, and caudate samples. The expression of *CNR1* is higher in fetal PFC and hippocampus, then drops down dramatically after birth. The lifespan trajectory of *CNR1* expression in the DLPFC differentially correlated with age by allelic variation at rs4680, a functional polymorphism in the *COMT* gene. Compared with *COMT* methionine^158^ carriers, Caucasian carriers of the *COMT* valine^158^ allele have a stronger negative correlation between the expression of *CNR1* in DLPFC and age. In contrast, the methylation level of cg02498983, which is negatively correlated with the expression of *CNR1* in PFC, showed the strongest positive correlation with age in PFC of Caucasian carriers of *COMT* valine^158^. Additionally, we have observed decreased mRNA expression of *CNR1* in the DLPFC of patients with schizophrenia. Further analysis revealed a positive eQTL SNP, rs806368, which predicted the expression of a novel transcript of *CNR1* in human DLPFC, hippocampus and caudate. This SNP has been associated with addiction and other psychiatric disorders. THC or ethanol are each significantly associated with dysregulated expression of *CNR1* in the PFC of patients with affective disorder, and the expression of *CNR1* is significantly upregulated in the PFC of schizophrenia patients who completed suicide. Our results support previous studies that have implicated the endocannabinoid system in the pathology of schizophrenia and provided additional insight into the mechanism of increasing risk for schizophrenia in the adolescent cannabis users.

## Introduction

Cannabis is a widely used and increasingly popular psychoactive substance for both recreational and medicinal purposes^1^, especially in Western countries^2^. However, cannabis use is not entirely benign. There is increasing evidence that it is associated with increased risk for psychosis^3^ including the recurrence of manic symptoms in patients with bipolar disorder. Adolescent cannabis use has been associated with a 2.4-fold increase in the risk of schizophrenia^4^, and early adolescent-onset use is associated with a higher risk^5^. One intriguing study found an increased risk for schizophrenia with early adolescent heavy usage, especially if the subjects had the allele encoding for valine in the *COMT* Val^158^Met polymorphism (rs4680)^6^. Cannabis use disorder is a common comorbidity in patients with schizophrenia, exacerbating psychosis, reducing neuroleptic efficacy, and increasing relapse rates^7–9^. Nevertheless, the vast majority of individuals who use cannabis never develop psychosis, suggesting that there may be genetic vulnerabilities to this adverse side effect. Interestingly, a recent study reported that an acute effect of ∆9-THC on brain physiology measured with fMRI in normal subjects was modulated by *COMT* Val^158^Met^10^.

Cannabis contains about 400 bioactive molecules, more than 60 of which are cannabinoid compounds^11^. The most psychoactive of these is delta-9-tetrahydocannabinol (∆9-THC), acting via the cannabinoid receptor type 1 (CB1) in the central nervous system (CNS). Delta-9-THC, as an exogenous agonist of endocannabinoid receptors, has been classified by some as a hallucinogen due to its effect on perception, especially in high doses and in individuals at high risk for mental illness^12^. Identification of the psychoactive components of cannabis led to the discovery of an important neurotransmitter system, the endocannabinoid system (ECS), comprised of cannabinoid receptors (CB1 and CB2), endogenous ligands (anandamide and 2-arachadonylogycerol), and their metabolic enzymes (fatty acid amide hydrolase and monoacylglycerol lipase)^13–18^. This system is widely distributed in the central and peripheral nervous systems and is implicated in a wide variety of brain functions, such as memory, mood, motor control, and reward processing^19–23^.

Although CB1 and CB2 are both G-protein-coupled receptors, they have different localization. The CB1 receptor, encoded by the *CNR1* gene, is expressed in both the central and peripheral nervous systems, especially on axon terminals in the cerebellum, hippocampus, basal ganglia, frontal cortex, amygdala, hypothalamus, and midbrain^24,25^. However, the CB2 receptor, encoded by the *CNR2* gene, is mainly expressed in the periphery, on cells in the immune system such as monocytes, macrophages, B-lymphocytes, and T-lymphocytes^13,26^, making the ECS potentially associated with immunomodulation^27,28^. The CB2 receptor is also expressed in the CNS, but mainly in microglia and other immune cells of brain^27,29,30^. The *CNR1* gene is composed of a single coding-exon and several alternative 5′ untranslated exons. The CB1 receptor, as expressed principally on presynaptic terminals, inhibits the release of excitatory and inhibitory neurotransmitters including acetylcholine, noradrenaline, 5-HT, GABA, glutamate, dopamine, D-aspartate, and cholecystokinin^31–34^. In summary, the CB1 receptor is an important component in the ECS in the nervous system, regulating synaptic transmission by modulating the release of neurotransmitters.

Genetic variants in *CNR1* have been reported to influence the clinical presentation of schizophrenia in patients exposed to cannabis. Heavy cannabis use in the context of certain *CNR1* genotypes may contribute to white matter volume deficits and cognitive impairment of patients with schizophrenia^35^. While some genetic studies have linked polymorphisms in the *CNR1* gene with increased risk of schizophrenia^36–38^, other studies have failed to find this association^39,40^. A significant increase in the level of the endocannabinoids has been observed in the cerebrospinal fluid of patients with schizophrenia^41^. There are also numerous reports of the altered cannabinoid receptor protein levels and receptor binding in patients with schizophrenia^42–44^. On balance, there is a growing body of literature implicating the ECS in schizophrenia.

To further characterize the molecular correlates underlying clinical associations of the ECS with psychosis in general and schizophrenia in particular, we measured the mRNA expression of *CNR1* across the human life span in the prefrontal cortex (PFC) and hippocampus and, based upon the Caspi study (2005)^6^, established expression profiles of the *CNR1* transcripts based on age and *COMT* genotype. We have also compared the expression of the *CNR1* transcripts in the PFC, hippocampus and caudate among controls, and individuals with either schizophrenia or affective disorders. To explore how epigenetics might affect the expression of *CNR1* transcripts, we measured DNA methylation profiles of *CNR1* in human PFC and hippocampus. By examining the relationship between the expression of *CNR1*, genetic variants and DNA methylation in a large human brain cohort across the lifespan and four different diagnostic groups, our study provides evidence of abnormalities in the ECS in association with schizophrenia, and defines expression quantitative trait loci and expression changes in this neuromodulatory system across the lifespan.

## Materials and methods

### Human postmortem brain tissue

Postmortem human brain tissue was collected at several sites for this study. A large number of samples were obtained at the Clinical Brain Disorders Branch (CBDB) at National Institute of Mental Health (NIMH) from the Northern Virginia and District of Columbia Medical Examiners’ Office, according to NIH Institutional Review Board guidelines (Protocol #90-M-0142). These samples were transferred to the Lieber Institute for Brain Development (LIBD) under an MTA with the NIMH. Additional samples were collected at the LIBD according to with a protocol approved by the Institutional Review Board of the State of Maryland Department of Health and Mental Hygiene (#12-24) and the Western Institutional Review Board (#20111080). Additional fetal, child, and adolescent brain tissue samples were provided by the National Institute of Child Health and Human Development Brain and Tissue Bank for Developmental Disorders via Material Transfer Agreements (NO1-HD-4-3368 and NO1-HD-4-3383) approved by Institutional Review Board of the University of Maryland. Audiotaped informed consent to study brain tissue was obtained for the legal next-of-kin on every case collected at NIMH and LIBD. Details of the donation process and specimens handling are described previously^45,46^. After next-of-kin provided audiotaped informed consent to brain donation, a standardized 36-item telephone screening interview was conducted, (the Lieber Institute for Brain Development Autopsy Questionnaire), to gather additional demographic, clinical, psychiatric history, substance abuse history, treatment, medical, and social history. A psychiatric narrative summary was written for every donor, to include data from multiple sources, including the Autopsy Questionnaire, medical examiner documents (investigative reports, autopsy reports, and toxicology testing), macroscopic and microscopic neuropathological examinations of the brain, as well as extensive psychiatric, detoxification, and medical record reviews, and/or supplemental family informant interviews using the MINI (Mini International Neuropsychiatric Interview). Two board-certified psychiatrists independently reviewed every case to arrive at DSM-5 lifetime psychiatric and substance use disorder diagnoses, including [schizophrenia and bipolar disorder, as well as substance abuse disorders], and if for any reason agreement was not reached between the two reviewers, a third board-certified psychiatrist was consulted. All donors were free from significant neuropathology, including cerebrovascular accidents and neurodegenerative diseases. Each subject was diagnosed retrospectively by two board-certified psychiatrists, according to the criteria in the DSM-IV. Brain specimens from the CBDB were transferred from the NIMH to the LIBD under a Material Transfer Agreement.

Available postmortem samples were selected based on RNA quality (RNA integrity number ≥ 5). In total, 703 DLPFC, 452 hippocampus, and 468 caudate postmortem brain samples were used in this study. The demographic data are summarized in Supplementary Table 1. In brief, the DLPFC cohort consists of 175 subjects with schizophrenia, 62 with bipolar disorder, 146 with major depression disorder (MDD), and 320 non-psychiatric controls. The hippocampus cohort includes 133 subjects with schizophrenia and 319 non-psychiatric controls. The caudate cohort contains 154 subjects with schizophrenia, 47 subjects with bipolar disorder and 273 non-psychiatric controls. The toxicological analysis was performed in each case. The non-psychiatric non-neurological controls had no known history of significant psychiatric or neurological illnesses, including substance abuse. Positive toxicology was exclusionary for control subjects but not for patients with psychiatric disorders.

### RNA extraction, RNA sequencing, and real-time PCR

Gray matter in the DLPFC, hippocampus and caudate were dissected out, pulverized and stored at −80 °C. Briefly, brains were hemisected, and cut into 1.0–1.5-cm-thick coronal slabs, flash-frozen, and stored at −80 °C. DLPFC gray matter was dissected using a dental drill. For non-fetal cases, the DLPFC (Brodmann’s areas 9 and 46) was dissected from the middle frontal gyrus of the coronal slab, immediately anterior to the genu of the corpus callosum. For fetal cases, the PFC was obtained from the frontal cortex dissected at the dorsal convexity, midway between the frontal pole and anterior temporal pole. The hippocampus was dissected from the anterior tip posteriorly through to the mid body of the hippocampus at the level of the lateral geniculate nucleus. For the fetal cases, the dorsal-medial aspect of the temporal lobe was removed, including the adjacent cortex, medial to the hippocampal sulcus. In the rostral-caudal axis, the dissection was performed at the mid-point of the Sylvian fissure. Caudate tissue was dissected from the head of the caudate nucleus, at the level of the nucleus accumbens. The dissection was restricted to the dorsal third of the caudate at this level, to make certain that this tissue was distinct from the adjacent nucleus accumbens. Total RNA was extracted from 30 to 50 mg of pulverized tissue with RNeasy Lipid Tissue Mini Kit (Qiagen). The RNA was purified with RNeasy Mini Spin columns with on-column DNase digestion by RNase-free DNase set (Qiagen). The yield of total RNA was determined by Qubit RNA BR Assay Kit and Qubit Fluorometer (ThermoFisher Scientific). The RNA quality was assessed with high-resolution capillary electrophoresis on an Agilent Bioanalyzer 2100 (Agilent Technologies). Approximately 300 ng total RNA was applied to an RNA 6000 Nano LabChip without prior heating. RNA integrity numbers (RIN) were obtained from the entire electrophoretic trace with the RIN software algorithm and was used for the assessment of RNA quality.

Poly-A RNA-seq for DLPFC and RiboZero RNA-seq for hippocampus and caudate were performed as described previously^47,48^. In general, Poly-A-containing RNA was purified from 1 μg DNase treated total RNA. Sequencing libraries were constructed by the Illumina TruSeq RNA Sample Preparation V2 kit. An index (up to 12) was inserted into Illumina adapters allowing samples to be multiplexed across lanes in each flow cell. After purification and enrichment, final cDNA libraries were sequenced by an Illumina HiSeq 2000 with paired-end 2 × 100 bp reads. For RiboZero RNA-seq, paired-end strand-specific sequencing libraries were constructed from 300 ng RNA using Illumina TruSeq Stranded Total RNA Library Preparation kit with Ribo-Zero Gold ribosomal RNA depletion. The libraries were sequenced on an Illumina HiSeq 3000. The Illumina Real-Time Analysis (RTA) module was used to perform image analysis and base calling. The BCL converter (CASAVA v1.8.2) was used to generate FASTQ files containing the sequencing reads. The sequencing reads were aligned to the human genome (UCSC hg19 build) using splicing-read mapper TopHat2^49^, STAR^50^ or GSNAP^51^, providing known transcripts from Ensembl Build GRCh37/hg19.

We have used real-time quantitative PCR (RT-PCR) to validate the mRNA expression of *CNR1* in 662 post-mortem DLPFC samples, consisting of 164 subjects with schizophrenia, 55 subjects with Bipolar disorder, 131 subjects with MDD, and 312 non-psychiatric controls (Supplementary Table 2). In short, 4 μg of total RNA was reverse transcribed by SuperScript First-Strand Synthesis System for RT-PCR (Invitrogen). mRNA expression levels of *CNR1* was measured by TaqMan Gene Expression Assay, Hs01038522_s1 (Applied Biosystems) using the ABI Prism 7900HT Sequence Detection System (Applied Biosystems). The mRNA expression level of *CNR1* in DLPFC was normalized to geometric means of two constitutively expressed genes: β-actin (ACTB) and β-glucuronidase (GUSB).

To measure the mRNA expression of the *CNR1* gene, we acquired the mapped reads covering the *CNR1* genomic region from BrainSeq datasets^48^, corresponding to chr6:88,849,585-88,875,767 on genome build GRCh37/hg19. Reads mapping to the gene was used to quantify the expression *CNR1* at the gene level. RPM (reads per million mapped reads in the targeted *CNR1* locus) has been calculated to quantify gene-level expression and junction-level expression.

### DNA extraction, genotyping, and DNA methylation

Genomic DNA was extracted from 100 mg of pulverized cerebellar tissue and DLPFC tissue with the phenol-chloroform method. SNP genotyping with HumanHap650Y, Human 1M-Duo BeadChips, HumanOmni5-Quad or HumanOmni2.5–8 (Illumina, San Diego, CA) was carried out according to the manufacturer’s instructions with DNA extracted from cerebellar tissue. The methylation and genotype data were extracted from a larger data set from one of our recent studies^52^. In brief, methylation of DNA extracted from DLPFC was assessed according to the manufacturer’s instruction using the Infinium HumanMethylation450 BeadChip Kit (Illumina), which measure DNA methylation of more than 485,000 CpG dinucleotides (cg) covering 99% RefSeq gene promoters, including the *CNR1* gene. The methylation data were processed and normalized using the *minfi* Bioconductor package in R^53^ as previously described^52^.

### Statistics

Statistical analyses were performed using the R package (Version3.5.2). The lifespan curve was generated using the LOESS fit (local polynomial regression fitting) by using an R package with default parameters. We controlled for heterogeneity of transcript feature expression resulting from potential latent factors by using principal component analysis (PCA). Expression principal components (PCs) were calculated using the log-transformed (log2 with an offset of 1) RPKM of all expressed transcript features mapping to the 2 Mb sequence flanking *CNR1* gene. Genomic PCs, which can model quantitative ethnicity (ancestry), were also calculated based on the genomic variants across common linkage-disequilibrium independent variants across the genome. The first ten expression PCs and the first five genetic PCs were used as covariates in the following eQTL analysis. Statistical analyses of mRNA expression associated with genotypes were conducted in the patients with schizophrenia and unaffected controls as our previous study (http://eqtl.brainseq.org/phase1/eqtl/)^47^. Levene’s test indicated that the assumption of homogeneity of variance was met. Statistical models investigating diagnosis-related expression patterns in adults (age > 13) was conducted by the general linear model (GLM), adjusted for ten expression PCs. Comparisons within the different diagnostic groups for completed suicide, nicotine exposure, ethanol exposure, and psychotropic medications (toxicology screen results about antidepressants, antipsychotics, anticonvulsants, benzodiazepines and opiates), lifetime neuroleptic exposure, average daily neuroleptic dose, final neuroleptic dose and illicit substances were also conducted by GLM adjusted for ten expression PCs. Estimated of lifetime neuroleptic exposure, average daily dose and final neuroleptic dose were all converted to chlorpromazine equivalents for statistical comparisons. Cannabis exposure was determined by the clinical history from next of kin interviews and medical record review, and toxicology screen, which measured Delta-9 THC, Delta-9 Carboxy THC and 11-Hydroxy Delta-9 THC. For qPCR validation, comparisons between the patient groups and controls were proceeded by GLM, adjusting for age, sex, race, and RIN. Pearson correlation was used to calculate the correlation between expression level or methylation level and age. The correlation coefficients were compared by Fisher’s r-to-z transformation.

## Results

**Fig. 1:**
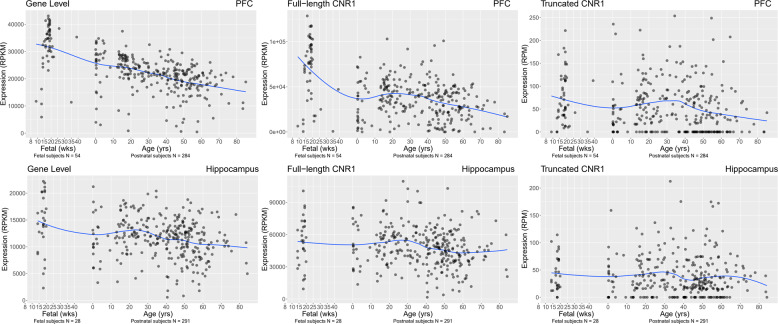
The mRNA expression lifetime trajectory of *CNR1* gene and *CNR1* transcripts in PFC and hippocampus of nonpsychiatric controls. The *x*-axis represents age. The *y*-axis represents the relative expression at gene level or transcript level in the DLPFC and hippocampus. Each dot represents a nonpsychiatric control subject.

Based on NCBI Reference Sequence Database Release 109 (hg38), there are 5 previously identified alternative transcripts of *CNR1* in human. These are predicted to produce only two different proteins: the previously identified full-length CB1 receptor and a truncated CB1 receptor. The truncated transcript is due to a 99 nt deletion located in the 5′ coding region of the last exon of *CNR1*, leading to a 439aa protein instead of the 472aa full-length CB1 receptor. We have used the reads aligning to the 99 nt deletion region and reads spanning the 99 nt deletion region to measure the expression of full-length and truncated *CNR1* transcripts respectively (Supplementary Fig. 1). Lifespan expression was measured from gestational weeks 14 to 20 and from birth up to 85 years of age in the DLPFC and hippocampus, and from birth to 86 years of age in caudate. In both PFC and hippocampus, the expression of *CNR1* at the gene level was the highest during the second-trimester fetal period, followed by decreased expression after birth (Fig. 1). The expression of the full-length and truncated *CNR1* transcripts showed the similar lifespan patterns in PFC, but the flat life-span expression trajectories in hippocampus (Fig. 1). The expression of the *CNR1* gene in PFC measured by RT-PCR also showed the same lifespan pattern (Supplementary Fig. 2). These findings suggest that the fetal brain may be susceptible to the effects of maternal cannabis consumption during pregnancy^54–56^.

### mRNA expression of *CNR1* in schizophrenia and affective disorders

**Fig. 2:**
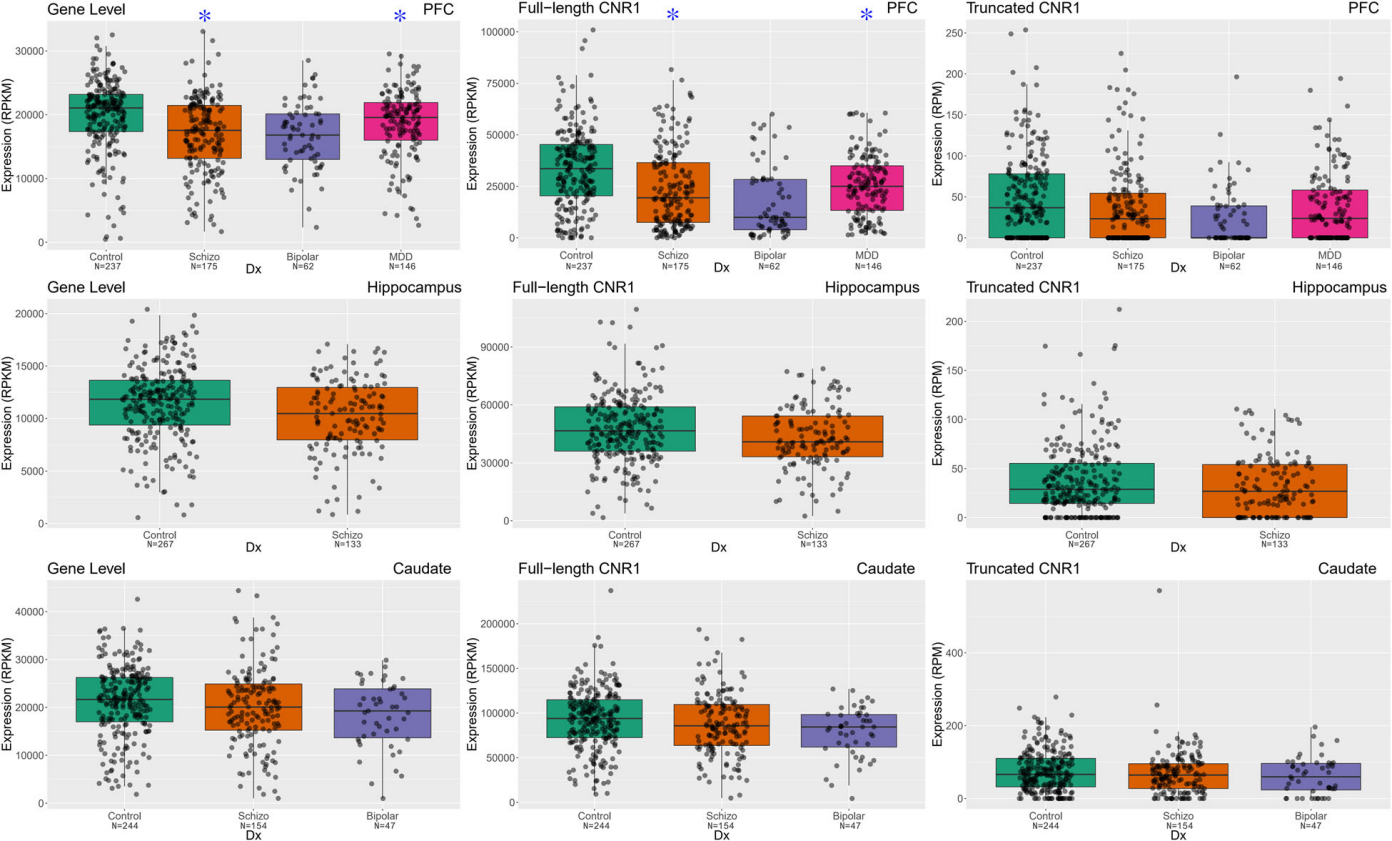
The comparison of mRNA expression of *CNR1* gene and *CNR1* transcripts in human DLPFC, hippocampus and caudate. The mRNA expression of *CNR1* gene, full-length *CNR1* transcript and 99 nt truncated *CNR1* transcript was studied across four cohorts of subjects in DLPFC, two cohorts of subjects in hippocampus and three cohorts of subjects in caudate. The *x*-axis showed the different diagnostic groups: Control, nonpsychiatric control group; Schizo, patients with schizophrenia; Bipolar, patients with bipolar disorder; MDD, patients with major depression. The *y*-axis represents the relative expression quantities at gene level or transcript level in the DLPFC, hippocampus or caudate. Each dot represents a subject. Blue stars indicate patient groups with significantly differential expression compared with control group.

We then examined the relationship between *CNR1* expression and diagnosis in neurotypical adult controls, and schizophrenia, bipolar, and major depression samples (age > 13). These analyses were conducted both at the gene and specific transcript level (full-length and truncated transcripts). In DLPFC, the expression of *CNR1* at the gene level (Fig. 2) is significantly decreased in patients with schizophrenia (*p* = 3.31E−04, FDR = 9.92E−04) and patients with MDD (*p* = 1.67E−04, FDR = 7.50E−04) compared with non-psychiatric controls, but not in patients with bipolar disorder (*p* = 5.49E−02). At the transcript level, the expression of the full-length *CNR1* transcript (Fig. 2) was downregulated significantly in the DLPFC of patients with schizophrenia (*p* = 8.86E−03, FDR = 0.02), and patients with MDD (*p* = 1.18E−04, FDR = 7.50E−04), but not in patients with bipolar disorder (*p* = 0.40). The expression of the truncated *CNR1* transcript (Fig. 2) was not significantly altered in the DLPFC of patients with schizophrenia (*p* = 0.62), bipolar disorder (*p* = 0.67) and MDD (*p* = 0.09).

There is regional selectively in the relationship between diagnosis and *CNR1* expression in the human brain. In the hippocampus, in contrast to the DLPFC, there is no significant difference in expression of the *CNR1* gene and transcripts between patients with schizophrenia and non-psychiatric controls (*p* > 0.5). Also, there is no significant difference between the expression of the *CNR1* gene and transcripts in caudate in the patients with schizophrenia or bipolar disorders, and non-psychiatric controls (*p* > 0.05).

To validate the expression difference revealed in DLPFC by RNA sequencing data, we performed TaqMan RT-PCR to measure the expression of the *CNR1* gene in postmortem PFC samples. By linear regression, the expression of the *CNR1 gene* is marginally decreased in the DLPFC of the patients with schizophrenia (*p* = 1.61E−02, FDR = 4.83E−02), but not in patients with bipolar disorder (*p* = 5.19E−02) or MDD (*p* = 0.81) (Supplementary Fig. 3). The RT-PCR assay (Hs01038522_s1) binds on the last exon of *CNR1*. The RT-PCR results partly validated the RNA sequencing findings. The different statistical significance might due to different cohorts, different covariates, or different reference loci of quantifications between RT-PCR and RNA sequencing.

**Fig. 3:**
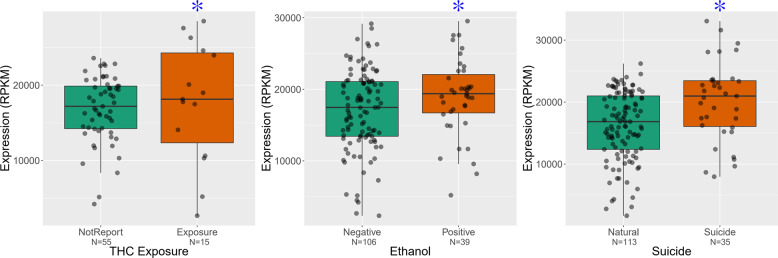
Expression of *CNR1* gene in PFC correlated with substance abuse and suicide. The *y*-axis represents the expression of *CNR1* gene in DLPFC of patients with schizophrenia and affective disorders. Each black dot represents a subject. THC or Ethanol exposure associated with the increased expression of *CNR1* gene in PFC with affective disorders, highlighted by blue stars. The *x*-axis showed the status of THC or ethanol exposure. The expression of *CNR1* gene increased in the PFC of patients with schizophrenia who completed suicide, highlighted by blue star. The x-axis separates suicide verses unsuicide schizophrenia patients.

Based on toxicology screens at the time of death and clinical records, we tested for an association between *CNR1* expression and death by completed suicide as well as illicit and legal drug exposure by GLM adjusted by the first ten expression PCs. Among the patients with affective disorders, the expression of *CNR1* gene in DLPFC is significantly upregulated in subjects with THC exposure (*p* = 4.13E−03, FDR = 0.025) or ethanol exposure (*p* = 5.23E−03, FDR = 0.03). The expression of the *CNR1* gene in DLPFC significantly increased in the patients with schizophrenia who had completed suicide (*p* = 0.01, FDR = 0.07) (Fig. 3). For a broad range of substances, such as nicotine, morphine, amphetamines, PCP, anticholinergics, anticonvulsants, antidepressants, antihistamines, anti-inflammatories, antipsychotics, CPZ equivalents, and lithium, there was no significant association with the expression of *CNR1* gene in PFC, hippocampus and caudate.

### Differential mRNA expression of *CNR1* in association with genetic variation in *COMT*

**Fig. 4:**
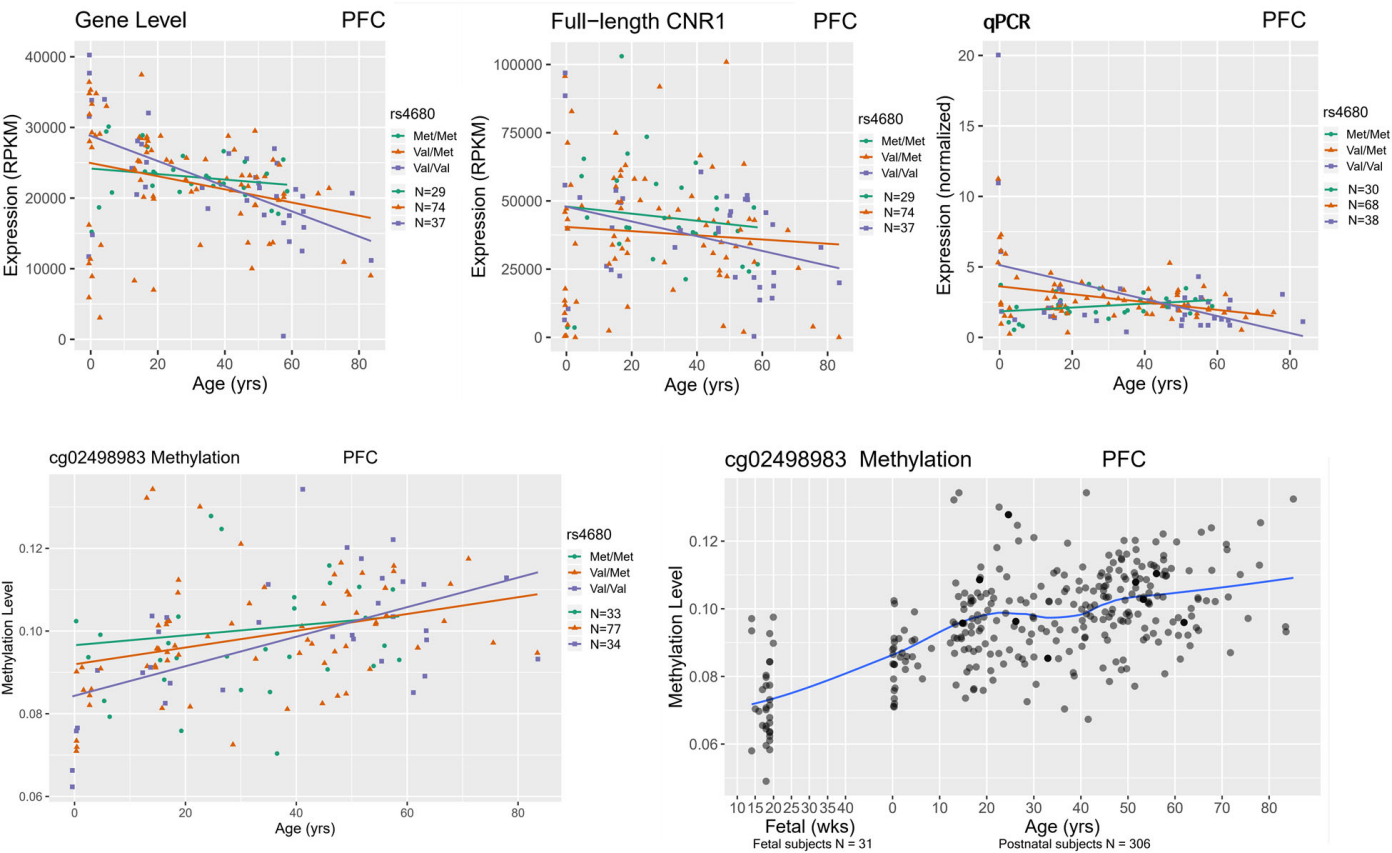
The mRNA expression of *CNR1* gene and methylation level of cg02498983 is differentially correlated with age in the PFC of Caucasian controls by rs4680 genotype. In the PFC of Caucasian controls who are *COMT* Val^158^ homozygotes, the expression of *CNR1* gene and full-length transcript is most negatively correlated with age. Our qPCR results also validated this finding. Interestingly, the methylation level of cg02498983 is most positively correlated with age in the PFC of Caucasian controls who are *COMT* Val^158^ homozygotes. Each dot represents a control subject: green line, rs4680 Met/Met genotype; orange line, rs4680 Val/Met genotype; purple line, rs4680 Val/Val genotype. Blue line represents the lifespan trajectory of the methylation level of cg02498983 in PFC.

To explore the interaction between rs4680, a functional polymorphism in the *COMT* gene, and early THC exposure, we hypothesized that the expression of *CNR1* in the human brain might have different developmental trajectories in association with rs4680 genotype. By Pearson’s correlation, the lifespan trajectory of *CNR1 gene* expression in PFC of Caucasian controls is differentially correlated with age by allelic variation at rs4680. Caucasian carriers of the *COMT* Val^158^ allele showed a significantly negative correlation between *CNR1* expression in PFC and age. The correlation coefficients were significantly different between the Caucasian carriers of the Val^158^ homozygotes and Met^158^ homozygotes (*p* = 0.01, FDR = 0.015). The Val^158^ homozygotes showed the steepest slope of the decline in expression of *CNR1* in PFC across the lifespan (COR = −0.55, *p* = 3.23E−04). The Val^158^Met heterozygotes also had a significantly negative slope of the expression of *CNR1* in PFC across the lifespan (COR = −0.44, *p* = 5.04E−05) (Fig. 4). At the transcript level, we observed similar correlations between the full-length *CNR1* transcript and age by rs4680 genotype (Fig. 4). In addition, the RT-PCR data validated that the Caucasian carriers of *COMT* Val^158^Val and Val^158^Met have negative correlations between the expression of full-length *CNR1* in DLPFC and age (COR = −0.42, *p* = 0.008; COR = −0.25, *p* = 0.004). This assay also validated the finding that Caucasian carriers with Val^158^ homozygote showed the steepest slope of expression of *CNR1* in PFC across the lifespan (COR = −0.42, *p* = 0.008) (Fig. 4), and the correlation coefficients were significantly different between the Caucasian carriers who were Val^158^ homozygotes compared with Met^158^ homozygotes (*p* = 6.11E−08, FDR = 1.45E−07).

By Pearson’s correlation, we have also observed that the lifespan trajectories of three methylation loci (cg19961480, cg08037684, and cg02498983) in the DLPFC of Caucasians are correlated with age by allelic variation at rs4680. Caucasian carriers of the *COMT* Val^158^ allele showed significantly positive correlations between methylation levels of these three loci in DLPFC and age (Table 1). Correlation coefficient comparisons showed that only cg02498983 has a significant slope difference comparing Caucasian Val^158^ homozygote carriers to Met^158^ homozygotes (*p* = 0.0042, FDR = 0.038). The Val^158^ homozygote Caucasian subjects had the steepest slope of methylation levels in PFC across the lifespan (Fig. 4). In the PFC of control subjects, these three methylation loci showed increased methylation level by age across the lifespan (Fig. 4, Supplementary Fig. 4), and significantly (*p* < E−20) negative correlations with the expression of *CNR1* gene and full-length *CNR1*.

**Table 1:** Pearson’s correlation between the methylation level of three loci and age in the PFC of Caucasians by genotype at rs4680, a functional polymorphism in the *COMT* gene.

	cg19961480		cg08037684		cg02498983	
rs4680	COR^a^	*p* value	COR^a^	*p* value	COR^a^	*p* value
Met/Met	0.16	8.83E−02	0.06	5.57E−01	0.23	1.73E−02
Val/Met	0.33	1.28E−06	0.29	2.62E−05	0.40	1.75E−09
Val/Val	0.36	4.71E−04	0.43	2.27E−05	0.60	3.35E−10

^a^COR represents the values of the Pearson Correlation.

### The association of genetic variation in *CNR1* at rs806368 with transcript expression in PFC, hippocampus and caudate

**Fig. 5:**
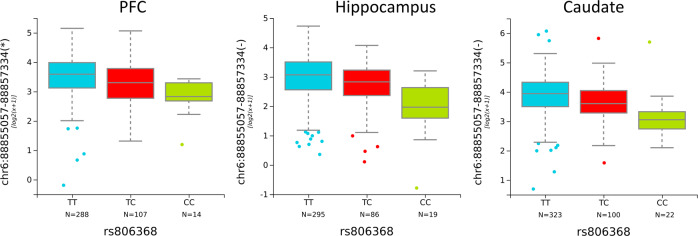
Positive eQTLs for *CNR1* with genetic variation at rs806368 in PFC, hippocampus, and caudate. rs806368 genotype significantly associated with the expression of a novel transcript in PFC, hippocampus and caudate. The *x*-axis showed the genotypes for each SNP. The *y*-axis represents the relative expression quantities in PFC, hippocampus or caudate.

For gene-level expression and full-length transcript expression of *CNR1*, we did not observe significant expression quantitative trait loci (eQTL). However, eQTL analyses for *CNR1* revealed a positive SNP, rs806368, which associated with expression of an alternative 5′ junction of *CNR1* (hg19: chr6:88855057-88857334). This unique junction links an alternative 5′ exon and the coding exon of *CNR1*, defining a novel *CNR1* transcript. This transcript was not previously identified in the RefSeq. rs806368 is located at the 3′ UTR of *CNR1*. Genetic variation at this locus predicted the expression of a novel transcript of *CNR1* in DLPFC (*p* = 8.42E−06, FDR = 9.75E−04) (Fig. 5), hippocampus (*p* = 4.46E−08, FDR = 1.87E−05) (Fig. 5) and caudate (*p* = 7.29E−08, FDR = 1.77E−05) (Fig. 5). In clinical studies, allelic variation at this SNP has been associated with increased risk for drug addiction, impulsivity, and nicotine addiction^57^. The clinically identified risk allele, C allele (minor allele), is associated with lower expression of the novel *CNR1* transcript. This novel transcript is characterized by a unique junction between the last coding exon and an alternative 5’ adjacent exon, which is 48 nt longer than the 143 nt canonical exon. In addition to rs806368, we have observed additional SNPs with statistically significant eQTLs for this newly identified novel *CNR1* transcript. However, these associations were found only in one or two brain regions, such as rs806371 in DLPFC (*p* = 3.61E−05, FDR = 6.19E−03), rs9450891 in hippocampus (*p* = 1.81E−05, FDR = 3.86E−03), or rs806371 in caudate (*p* = 5.45E−07, FDR = 1.09E−04) (Supplementary Table 3).

## Discussion

Brain maturation continues during adolescence, especially in the limbic and neocortical regions involved in the regulation of complex behaviors and emotional responses. Multiple studies have suggested that changes in the ECS during adolescence contribute to the maturation of local corticocortical and corticolimbic circuit neurons^58,59^. Changes in the ECS during adolescence may produce long-lasting effects into adulthood. Caspi et al.^6^ presented evidence that cannabis exposure during adolescence may interact with genetic factors, such as *COMT* genotype at rs4680, to impact brain maturation and the risk for emergence of psychotic symptoms.

The CB1 receptor is the main cannabinoid receptor in the brain, and evidence in animal and human studies have suggested that dysregulation of the CB1 receptor in multiple brain regions, including the DLPFC and hippocampus, is involved in the development of schizophrenia by compromising complex circuits mediating cognition and memory^60^. In this study, we measured mRNA expression of the *CNR1* gene, which encodes the CB1 receptor, across the human life span in the PFC and hippocampus, and established the expression profiles of *CNR1* gene based on age and *COMT* genotype. Our results support previous studies that suggested a link between *CNR1* and *COMT* genotype. In particular, the expression of *CNR1* was the highest during the fetal period, followed by decreased expression after birth in human PFC and hippocampus. The correlations between *CNR1* expression in the PFC of Caucasians and age differ by *COMT* genotype. Moreover, we have observed that the methylation levels at locus, cg02498983, is differentially correlated with age by *COMT* genotype in the PFC of Caucasians. In Caucasians, the *COMT* Val^158^Val carriers have the steepest negative slope of *CNR1* expression and positive slope of methylation levels in PFC across lifespan compared with *COMT* Met^158^Val and Met^158^Met carriers. These results suggest that the levels of CNR1 protein might mediate the vulnerability towards dysmaturation of the PFC, secondary to cannabis exposure during adolescence. *CNR1* expression levels in the PFC are modulated by rs4680 genotype in Caucasians, thereby altering signaling through the ECS. This in turn may lead to abnormal circuit formation between the PFC and other brain regions, including the hippocampal formation, the key elements for increasing risk of psychotic symptoms in adolescent cannabis users. During the development, CB1 receptor is critical to regulate neurotransmitter release and synaptic plasticity^61^, as well as neurodevelopmental programming for various of events such as proliferation, neurite growth and synaptogenesis^62^. Excessive stimulation of the cannabinoid system with exogenous cannabis during brain development, including in the adolescent period, may alter the biology and function of critical components of the maturing brain. This is especially important in the maturation of prefrontal regions of the brain as significant maturational events are not completed until early adulthood. It is not surprising that abnormal development and connectivity of the PFC as a result of cannabis exposure in genetically vulnerable individuals may lead to long-term effects on brain function later in life. Animal studies have suggested that maternal cannabis use affects the development of neural circuits, especially the dopaminergic system. In addition, animal model studies have found behavioral and cognitive deficits caused by cannabinoid exposure during early developmental periods^63^. These deficits are associated with changes of neurotransmission, especially in hippocampal and cortical excitatory circuits^63^. This disparity between different genotype groups is an example of different genetic backgrounds leading to a differential impact on gene expression and possibly differential susceptibility to the impact of environmental factors. We have also tested other genes they are important components of the cannabinoid signaling system, such as *CNR2*, *FAAH* and *MGLL* (Supplementary Fig. 5), but did not observe any positive associations between rs4680 genotype and gene expression in human postmortem brains.

The last exon of the *CNR1* gene is transcribed to produce the CB1 receptor. Previous studies have reported several splicing variants of *CNR1* with a truncated coding sequence in humans^64,65^, caused by several deletions inside the last exon of *CNR1*. Current RefSeq maps show one well-characterized splicing variant for *CNR1* with a truncated coding sequence (NM_033181.3), caused by an in-exon 99 nt deletion. Splicing variants with truncated coding sequences are rare in the human PFC^66^, hippocampus and caudate. There are additional splicing events in the 5’ UTR of the *CNR1* mRNA, but these do not change the protein-coding sequence. The longest 5′ UTR in *CNR1* is about 500 nt and contains more than 600 potential transcription factor binding sites for 153 distinct transcription factors^67^. In this study, we have reported a novel junction in the 5’ UTR, which introduced an extra 48 nt sequence into the 5’ UTR. This novel extra 48 nt sequence contains an estimated 40 potential transcription factor binding sites (Supplementary Table 4). Three of them are unique and will bind Signal Transducer and Activator of Transcription proteins, whose biological activities ultimately regulate many critical aspects of cell growth, survival, and differentiation. The complexity of the 5′ UTR of *CNR1* gene suggests that the expression of the *CNR1* gene is regulated by multiple transcription factors in an “on-demand” fashion and this regulation may be important in aspects of brain development and function.

Our finding of decreased *CNR1* expression in schizophrenia replicates and extends findings from previous studies. Egan et al. reported decreases in both CB1 mRNA and receptor levels, measured by in situ hybridization and immunocytochemistry, in the DLPFC of patients with schizophrenia^68^. A more recent study reported the lower availability of CB1 receptor in male patients with first-episode psychosis, greater reductions in CB1 receptor levels were associated with poor cognitive functioning and symptom severity^69^. Previously our group reported decreased expression of CB1 in layer 6 of the PFC of patients with schizophrenia, using in situ hybridization^70^. Consistent with our previous study^70^, here we have found decreased expression of the *CNR1* gene in the DLPFC of patients with schizophrenia compared with non-psychiatric controls. In short, in examining three brain regions from one of the largest postmortem cohorts to date, our study has observed lower expression of *CNR1* in DLPFC of patients with schizophrenia. To be certain, although *CNR1* expression can be modulated pharmacologically by a large number of compounds, including atypical antipsychotic medications^71^, the results in this study are not clearly affected by those potential confounds. It is worth noting that, if we applied qSVA framework^72^ for removing confounding effects instead of expression PCs, there are no significant difference of the expression of CNR1 between the control group and case groups.

An important confound for postmortem human brain studies is comorbid substance use disorders. Comparisons of affective disorder patients with and without THC or ethanol exposure revealed upregulated expression of the *CNR1* gene in the patients with mood disorders. In light of this finding we then examined a SNP at rs806368. Genotype at this locus in clinical studies has been associated with increased risk of cocaine dependence^73,74^, alcohol dependence^75,76^, nicotine dependence^77^, cannabis dependence^78^, impulsivity^79^, and drug dependence^76^. Here we have found that the minor allele of rs806368, a risk allele for substance dependence, is associated with lower expression of a novel *CNR1* transcript in DLPFC. Our results provide further evidence that dysregulation of endocannabinoid signaling may be involved in substance abuse disorders, at least in patients with comorbid mood disorders. Since the novel transcript produces the same canonical CB1 receptor despite its unique 5’ UTR, it is more likely that this novel splicing event regulates the expression level of CB1 receptor in response to specific environmental stimuli, such as substance abuse. To understand the underlying mechanism of this positive eQTL, it is necessary to characterize this transcript at the cellular level across human development and to study this transcript in animal models of substance abuse and addiction.

There is abundant evidence from rodents and humans indicating that endocannabinoid signaling undergoes age-dependent changes^80^. CB1 receptor expression, its coupling to G proteins and the level of 2-Arachidonoylglycerol (2-AG) are reduced in the brain of older animals^81–84^. Fatty acid amide hydrolase (FAAH) is the primary catabolic enzyme of anandamide (AEA), which is one of two endogenous cannabinoid ligands^85^. The mRNA and protein levels and protein activity of FAAH are increased in late-onset (age > 65) Alzheimer’s disease (LOAD) patients^86^. Low doses of THC can reverse the age-related decline in cognitive performance in older animals, accompanied by enhanced expression of synaptic markers and increased hippocampal spine density^87^. In summary, the activity of the ECS declines during aging with decreased receptor levels and activity and decreased levels of an endogenous cannabinoid ligand because of increased levels of its catabolizing enzyme. In this study, we showed that the decreased lifespan trajectory of mRNA expression of *CNR1* in PFC and hippocampus, as well as the increased lifespan trajectory the methylation level of cg02498983 in PFC. Our data suggested the decreasing signaling of cannabinoid system during the aging in the human brain, especially PFC.

There are high rates of recreational and illicit drug use among patients with schizophrenia and mood disorders^88–92^. Explanations include shared genetic vulnerabilities, self-medication to mitigate the side effects of medications, and/or psychosocial factors^93,94^. The self-medication hypothesis proposes that self-administered substances may reduce negative symptoms. As a consequence, positive symptoms appear to be more prominent among substance-abusing patients with schizophrenia. Genetic studies have observed positive associations between *CNR1* gene and substance abuse, including cannabis, alcohol, nicotine, and cocaine^73,76,77,95–97^. The CB1 receptor regulates the release of neurotransmitters, modulating neuronal activity throughout the CNS^98,99^. In patients with schizophrenia, cigarette smokers require significantly higher neuroleptic doses, and smoking is associated with a significant reduction in measurements of medication-induced Parkinsonism^100^. Possibly, increasing activity of ECS normalizes neurotransmission in multiple neural networks in the CNS thereby decreasing both negative symptoms and the discomforting Parkinsonian side effects causing by antipsychotic medications. Our results suggest that allelic variation in the *CNR1* gene is associated with differential gene expression, leading to differential vulnerability towards the development of psychiatric disorders. As shown by our findings, abnormalities in mRNA expression in postmortem human brains support the hypothesis that dysregulation of the ECS is involved in the pathology of schizophrenia and affective disorders. It follows that the ECS is a potential therapeutic target for both schizophrenia and affective disorders.

In conclusion, we have found differential expression of *CNR1* in PFC and caudate of patients with psychosis, namely schizophrenia, bipolar disorder, and MDD. While *CNR1* expression declines overall with aging, there is a differential effect of *COMT* genotype on age-related gene expression in Caucasians. We also have identified differences in methylation in *CNR1* by *COMT* genotype, which represent the epigenetic contributions of the environment to *CNR1* expression in the course of normal human brain development and psychosis. And last, but not least, we have identified a novel transcript in *CNR1*, whose expression is associated with a genetic variant that also is associated with increased risk for drug addiction, a major comorbidity in both schizophrenia and affective disorders.

## Supplementary information

Supplementary Figure Legends

Supplementary Table 1

Supplementary Table 2

Supplementary Table 3

Supplementary Table 4

Supplementary Figure 1

Supplementary Figure 2

Supplementary Figure 3

Supplementary Figure 4

Supplementary Figure 5
