# Supplementary material for: Cannabinoid receptor CNR1 expression and DNA methylation in human prefrontal cortex, hippocampus and caudate in brain development and schizophrenia
Source: Transl Psychiatry. 2020 May 19;10:158. doi: 10.1038/s41398-020-0832-8 (PMC7237456; doi:10.1038/s41398-020-0832-8)
Supplement: Supplementary file 1 — Supplementary Figure Legends [file 41398_2020_832_MOESM1_ESM.docx]

**Supplementary Figure Legends**

**Supplementary Figure 1.** The gene structure of CNR1, as shown in the UCSC Genomic Browser. Black and blue bars represent exons, and thick bars represent code portion of exons. The arrows indicate the direction of the gene (From 5’ to 3’).

**Supplementary Figure 2.** The mRNA expression lifetime trajectory of CNR1 gene in PFC by qPCR. The x-axis represents age. The y-axis represents the relative expression quantities of CNR1 in the DLPFC. Each dot represents a nonpsychiatric control subject.

**Supplementary Figure 3.** The mRNA expression of CNR1 gene was studied by qPCR across four cohorts of subjects in DLPFC. The x-axis showed the different diagnostic groups: Control: nonpsychiatric control group; Schizo: patients with schizophrenia; Bipolar: patients with bipolar disorder; MDD: patients with major depression. The y-axis represents the relative expression quantities at gene level or transcript level in the DLPFC, hippocampus or caudate. Each dot represents a subject. Blue star indicates patient groups with significantly differential expression compared with control group.

**Supplementary Figure 4.** The methylation lifetime trajectory of cg08037684 and cg19961480 in human PFC. The x-axis represents age. The y-axis represents the relative DNA methylation level in the DLPFC. Each dot represents a nonpsychiatric control subject. Blue line represents the lifespan trajectory of the methylation level in PFC.

**Supplementary Figure 5.** The mRNA expression lifetime trajectory of CNR2, FAAH and MGLL gene in PFC and hippocampus of nonpsychiatric controls. The x-axis represents age. The y-axis represents the relative expression at gene level or transcript level in the DLPFC and hippocampus. Each dot represents a nonpsychiatric control subject.
