# Supplementary material for: Cannabinoid receptor CNR1 expression and DNA methylation in human prefrontal cortex, hippocampus and caudate in brain development and schizophrenia
Source: Transl Psychiatry. 2020 May 19;10:158. doi: 10.1038/s41398-020-0832-8 (PMC7237456; doi:10.1038/s41398-020-0832-8)
Supplement: Supplementary file 2 — Supplementary Table 1 [file 41398_2020_832_MOESM2_ESM.docx]

**Supplementary Table 1. Demographic information of human postmortem samples (RNA Sequencing)**

| **Cohort** | **Number** | **Sex** | **Race** | **Age** | **PMI(h)** | **pH** | **RIN** |
| --- | --- | --- | --- | --- | --- | --- | --- |
| **DLPFC cohort** |  |  |  |  |  |  |  |
| Controls (age<13) | 83 | 49M/34F | 51AA/32CAUC | 0.8±2.6 | 13.5±16.1 | 6.4±0.3 | 8.5±1.2 |
| Controls (age>13) | 237 | 166M/71F | 124AA/113CAUC | 40.3±17.3 | 28.8±14.8 | 6.5±0.3 | 8.3±0.9 |
| SZ patients | 175 | 111M/64F | 79AA/96CAUC | 49.7±14.7 | 38.1±23.6 | 6.4±0.3 | 7.9±0.9 |
| BP patients | 62 | 33M/29F | 6AA/56CAUC | 45.7±14.4 | 31.8±17.9 | 6.3±0.3 | 7.8±1.0 |
| MDD patients | 146 | 87M/59F | 15AA/131CAUC | 44.1±13.7 | 36.6±25.1 | 6.4±0.3 | 8.0±0.9 |
| **Hippocampus cohort** |  |  |  |  |  |  |  |
| Controls (age<13) | 52 | 27M/25F | 35AA/17CAUC | 0.9±2.7 | 14.3±16.3 | 6.4±0.3 | 8.4±1.4 |
| Controls (age>13) | 267 | 186M/81F | 142AA/125CAUC | 42.7±16.2 | 30.1±13.8 | 6.5±0.3 | 7.7±1.0 |
| SZ patients | 133 | 86M/47F | 74AA/59CAUC | 51.5±15.3 | 36.2±17.7 | 6.4±0.3 | 7.3±1.1 |
| **Caudate cohort** |  |  |  |  |  |  |  |
| Controls (age<13) | 23 | 16M/7F | 12AA/11CAUC | 1.3±2.1 | 32.3±16.2 | 6.3±0.2 | 7.2±1.1 |
| Controls (age>13) | 244 | 167M/77F | 124AA/120CAUC | 46.8±16.2 | 31.0±13.0 | 6.5±0.3 | 7.9±0.8 |
| SZ patients | 154 | 103M/51F | 85AA/69CAUC | 51.6±15.1 | 36.1±20.5 | 6.4±0.2 | 7.8±1.0 |
| BP patients | 47 | 28M/19F | 4AA/43CAUC | 43.7±12.3 | 29.5±16.5 | 6.3±0.3 | 7.7±0.9 |

*AA, African American; CAUC, Caucasian; AS, Asian; HISP, Hispanic; F, Female; M, Male; SZ, Schizophrenia; BP, Bipolar Disorder; MDD, Major Depressive Disorder; PMI, Postmortem Interval; RIN, RNA Integrity Number.*
