# Supplementary material for: Cannabinoid receptor CNR1 expression and DNA methylation in human prefrontal cortex, hippocampus and caudate in brain development and schizophrenia
Source: Transl Psychiatry. 2020 May 19;10:158. doi: 10.1038/s41398-020-0832-8 (PMC7237456; doi:10.1038/s41398-020-0832-8)
Supplement: Supplementary file 3 — Supplementary Table 2 [file 41398_2020_832_MOESM3_ESM.docx]

**Supplementary Table 2. Demographic information of human postmortem samples (qPCR)**

| **Cohort** | **Number** | **Sex** | **Race** | **Age** | **PMI(h)** | **pH** | **RIN** |
| --- | --- | --- | --- | --- | --- | --- | --- |
| **DLPFC cohort** |  |  |  |  |  |  |  |
| Controls (age<13) | 81 | 46M/35F | 55AA/26CAUC | 1.0±2.8 | 15.1±16.8 | 6.4±0.3 | 8.4±1.3 |
| Controls (age>13) | 231 | 160M/71F | 121AA/110CAUC | 39.7±17.4 | 28.5±14.2 | 6.5±0.3 | 8.3±0.7 |
| SZ patients | 164 | 103M/61F | 70AA/94CAUC | 50.3±15.0 | 38.4±23.9 | 6.4±0.3 | 7.8±1.0 |
| BP patients | 55 | 32M/23F | 4AA/51CAUC | 43.9±14.2 | 33.3±18.7 | 6.3±0.3 | 8.0±0.9 |
| MDD patients | 131 | 76M/55F | 14AA/117CAUC | 45.4±14.2 | 38.4±25.8 | 6.4±0.3 | 8.0±0.9 |

*AA, African American; CAUC, Caucasian; AS, Asian; HISP, Hispanic; F, Female; M, Male; SZ, Schizophrenia; BP, Bipolar Disorder; MDD, Major Depressive Disorder; PMI, Postmortem Interval; RIN, RNA Integrity Number.*
