# Supplementary material for: Cannabinoid receptor CNR1 expression and DNA methylation in human prefrontal cortex, hippocampus and caudate in brain development and schizophrenia
Source: Transl Psychiatry. 2020 May 19;10:158. doi: 10.1038/s41398-020-0832-8 (PMC7237456; doi:10.1038/s41398-020-0832-8)
Supplement: Supplementary file 4 — Supplementary Table 3 [file 41398_2020_832_MOESM4_ESM.docx]

Supplementary Table 3. Other eQTLs of CNR1 gene in human DLPFC, Hippocampus and Caudate

Part I. DLPFC

| SNP | Feature | Type | beta | pvalue | FDR | bonf | snpRsNum | Coordinates | Symbol | EnsemblID | Class | Ref | Counted |
| --- | --- | --- | --- | --- | --- | --- | --- | --- | --- | --- | --- | --- | --- |
| rs806368 | e407431 | Exon | -0.1499 | 8.747E-06 | 0.001008 | 1 | rs806368 | chr6:88857383-88857525(-) | CNR1 | ENSG00000118432 | InEns | T | C |
| rs1090711:88803767:T:C | chr6:88855057-88876684(*) | Junction | -0.295 | 8.785E-06 | 0.001829 | 1 | rs1090711 | chr6:88855057-88876684(*) | CNR1 | ENSG00000118432 | AltStartEnd | C | T |
| rs806368 | chr6:88855057-88857334(*) | Junction | -0.3153 | 9.644E-06 | 0.001984 | 1 | rs806368 | chr6:88855057-88857334(*) | CNR1 | ENSG00000118432 | AltStartEnd | T | C |
| rs76411302:88944301:A:G | ENSG00000118432 | Gene | 0.111 | 0.00001725 | 0.001304 | 1 | rs76411302 | chr6:88849583-88876078(-) | CNR1 | ENSG00000118432 | InEns | A | G |
| rs1090713:88804675:C:T | chr6:88855057-88876684(*) | Junction | -0.2538 | 0.00001962 | 0.003672 | 1 | rs1090713 | chr6:88855057-88876684(*) | CNR1 | ENSG00000118432 | AltStartEnd | T | C |
| rs76411302:88944301:A:G | e407413 | Exon | 0.1351 | 0.00002111 | 0.002172 | 1 | rs76411302 | chr6:88849583-88855056(-) | CNR1 | ENSG00000118432 | InEns | A | G |
| rs76411302:88944301:A:G | e407419 | Exon | 0.1351 | 0.00002114 | 0.002174 | 1 | rs76411302 | chr6:88849595-88855056(-) | CNR1 | ENSG00000118432 | InEns | A | G |
| rs6454650:88518960:G:C | TCONS_00584635 | Transcript | 0.1284 | 0.00002321 | 0.007916 | 1 | rs6454650 | chr6:88849583-88875767(-) | CNR1 | ENSG00000118432 | InEns | C | G |
| rs9359758:88518581:G:T | TCONS_00584635 | Transcript | 0.1277 | 0.00002525 | 0.008491 | 1 | rs9359758 | chr6:88849583-88875767(-) | CNR1 | ENSG00000118432 | InEns | T | G |
| rs57743608:88941889:G:A | e407413 | Exon | 0.1275 | 0.00003348 | 0.003231 | 1 | rs57743608 | chr6:88849583-88855056(-) | CNR1 | ENSG00000118432 | InEns | G | A |
| rs57743608:88941889:G:A | e407419 | Exon | 0.1275 | 0.00003353 | 0.003235 | 1 | rs57743608 | chr6:88849595-88855056(-) | CNR1 | ENSG00000118432 | InEns | G | A |
| rs150832842:88934311:TC:T | e407413 | Exon | 0.1274 | 0.00003376 | 0.003254 | 1 | rs570118227 | chr6:88849583-88855056(-) | CNR1 | ENSG00000118432 | InEns | TC | T |
| rs7768339:88932459:G:C | e407413 | Exon | 0.1274 | 0.00003376 | 0.003254 | 1 | rs7768339 | chr6:88849583-88855056(-) | CNR1 | ENSG00000118432 | InEns | G | C |
| rs150832842:88934311:TC:T | e407419 | Exon | 0.1274 | 0.00003381 | 0.003258 | 1 | rs570118227 | chr6:88849595-88855056(-) | CNR1 | ENSG00000118432 | InEns | TC | T |
| rs7768339:88932459:G:C | e407419 | Exon | 0.1274 | 0.00003381 | 0.003258 | 1 | rs7768339 | chr6:88849595-88855056(-) | CNR1 | ENSG00000118432 | InEns | G | C |
| rs806371:88856363:T:G | chr6:88855057-88857334(*) | Junction | -0.2929 | 0.00003614 | 0.006189 | 1 | rs806371 | chr6:88855057-88857334(*) | CNR1 | ENSG00000118432 | AltStartEnd | T | G |
| rs57743608:88941889:G:A | ENSG00000118432 | Gene | 0.103 | 0.00003766 | 0.002577 | 1 | rs57743608 | chr6:88849583-88876078(-) | CNR1 | ENSG00000118432 | InEns | G | A |
| rs73752455:88924705:G:A | e407413 | Exon | 0.1274 | 0.00003853 | 0.003645 | 1 | rs73752455 | chr6:88849583-88855056(-) | CNR1 | ENSG00000118432 | InEns | G | A |
| rs73752455:88924705:G:A | e407419 | Exon | 0.1274 | 0.00003856 | 0.003647 | 1 | rs73752455 | chr6:88849595-88855056(-) | CNR1 | ENSG00000118432 | InEns | G | A |
| rs7768339:88932459:G:C | ENSG00000118432 | Gene | 0.1027 | 0.00003919 | 0.002669 | 1 | rs7768339 | chr6:88849583-88876078(-) | CNR1 | ENSG00000118432 | InEns | G | C |
| rs150832842:88934311:TC:T | ENSG00000118432 | Gene | 0.1027 | 0.00003919 | 0.002669 | 1 | rs570118227 | chr6:88849583-88876078(-) | CNR1 | ENSG00000118432 | InEns | TC | T |
| rs116403728:88913535:A:G | e407413 | Exon | 0.127 | 0.00004042 | 0.003798 | 1 | rs78041938 | chr6:88849583-88855056(-) | CNR1 | ENSG00000118432 | InEns | A | G |
| rs55773232:88914262:C:A | e407413 | Exon | 0.127 | 0.00004042 | 0.003798 | 1 | rs55773232 | chr6:88849583-88855056(-) | CNR1 | ENSG00000118432 | InEns | C | A |
| rs7743518:88916868:G:T | e407413 | Exon | 0.127 | 0.00004042 | 0.003798 | 1 | rs7743518 | chr6:88849583-88855056(-) | CNR1 | ENSG00000118432 | InEns | G | T |
| rs7775243 | e407413 | Exon | 0.127 | 0.00004042 | 0.003798 | 1 | rs7775243 | chr6:88849583-88855056(-) | CNR1 | ENSG00000118432 | InEns | A | G |
| rs75168758:88915443:T:A | e407413 | Exon | 0.127 | 0.00004042 | 0.003798 | 1 | rs75168758 | chr6:88849583-88855056(-) | CNR1 | ENSG00000118432 | InEns | T | A |
| rs75168758:88915443:T:A | e407419 | Exon | 0.1271 | 0.00004045 | 0.0038 | 1 | rs75168758 | chr6:88849595-88855056(-) | CNR1 | ENSG00000118432 | InEns | T | A |
| rs7775243 | e407419 | Exon | 0.1271 | 0.00004045 | 0.0038 | 1 | rs7775243 | chr6:88849595-88855056(-) | CNR1 | ENSG00000118432 | InEns | A | G |
| rs55773232:88914262:C:A | e407419 | Exon | 0.1271 | 0.00004045 | 0.0038 | 1 | rs55773232 | chr6:88849595-88855056(-) | CNR1 | ENSG00000118432 | InEns | C | A |
| rs7743518:88916868:G:T | e407419 | Exon | 0.1271 | 0.00004045 | 0.0038 | 1 | rs7743518 | chr6:88849595-88855056(-) | CNR1 | ENSG00000118432 | InEns | G | T |
| rs116403728:88913535:A:G | e407419 | Exon | 0.1271 | 0.00004045 | 0.0038 | 1 | rs78041938 | chr6:88849595-88855056(-) | CNR1 | ENSG00000118432 | InEns | A | G |
| rs73752455:88924705:G:A | ENSG00000118432 | Gene | 0.1026 | 0.0000459 | 0.003061 | 1 | rs73752455 | chr6:88849583-88876078(-) | CNR1 | ENSG00000118432 | InEns | G | A |
| rs55773232:88914262:C:A | ENSG00000118432 | Gene | 0.1023 | 0.00004836 | 0.003202 | 1 | rs55773232 | chr6:88849583-88876078(-) | CNR1 | ENSG00000118432 | InEns | C | A |
| rs75168758:88915443:T:A | ENSG00000118432 | Gene | 0.1023 | 0.00004836 | 0.003202 | 1 | rs75168758 | chr6:88849583-88876078(-) | CNR1 | ENSG00000118432 | InEns | T | A |
| rs7775243 | ENSG00000118432 | Gene | 0.1023 | 0.00004836 | 0.003202 | 1 | rs7775243 | chr6:88849583-88876078(-) | CNR1 | ENSG00000118432 | InEns | A | G |
| rs116403728:88913535:A:G | ENSG00000118432 | Gene | 0.1023 | 0.00004836 | 0.003202 | 1 | rs78041938 | chr6:88849583-88876078(-) | CNR1 | ENSG00000118432 | InEns | A | G |
| rs7743518:88916868:G:T | ENSG00000118432 | Gene | 0.1023 | 0.00004836 | 0.003202 | 1 | rs7743518 | chr6:88849583-88876078(-) | CNR1 | ENSG00000118432 | InEns | G | T |
| rs2756373:89297642:G:A | e407423 | Exon | 0.1745 | 0.00007998 | 0.006774 | 1 | rs2756373 | chr6:88854932-88854993(-) | CNR1 | ENSG00000118432 | InEns | G | A |
| rs55667151:88909835:G:A | ENSG00000118432 | Gene | 0.09822 | 0.00008971 | 0.005444 | 1 | rs55667151 | chr6:88849583-88876078(-) | CNR1 | ENSG00000118432 | InEns | G | A |
| rs150254251:88943980:C:CAA | ENSG00000118432 | Gene | 0.07729 | 0.00013 | 0.007475 | 1 | rs578257573 | chr6:88849583-88876078(-) | CNR1 | ENSG00000118432 | InEns | C | CAA |

Part II. Hippocampus

| SNP | Feature | Type | beta | pvalue | FDR | bonf | snpRsNum | Coordinates | Symbol | EnsemblID | Class | Ref | Counted |
| --- | --- | --- | --- | --- | --- | --- | --- | --- | --- | --- | --- | --- | --- |
| rs806368 | chr6:88855057-88857334(-) | Junction | -0.4291 | 4.46E-08 | 0.00001868 | 1 | rs806368 | chr6:88855057-88857334 | CNR1 | ENSG00000118432 | AltStartEnd | T | C |
| rs9450891:88830721:C:T | chr6:88855057-88857334(-) | Junction | 0.2571 | 0.00001805 | 0.003862 | 1 | rs9450891 | chr6:88855057-88857334 | CNR1 | ENSG00000118432 | AltStartEnd | C | T |
| rs10944343:88831383:C:T | chr6:88855057-88857334(-) | Junction | 0.2529 | 0.00002866 | 0.00572 | 1 | rs10944343 | chr6:88855057-88857334 | CNR1 | ENSG00000118432 | AltStartEnd | C | T |
| rs138741534:88675030:A:AG | chr6:88857526-88876684(-) | Junction | 0.2367 | 0.00002965 | 0.005887 | 1 | rs535077359 | chr6:88857526-88876684 | CNR1 | ENSG00000118432 | AltStartEnd | A | AG |
| rs9353524:88837616:C:G | chr6:88855057-88857334(-) | Junction | -0.3001 | 0.0001387 | 0.02108 | 1 | rs9353524 | chr6:88855057-88857334 | CNR1 | ENSG00000118432 | AltStartEnd | C | G |

Part III. Caudate

| SNP | Feature | Type | beta | pvalue | FDR | bonf | snpRsNum | Coordinates | Symbol | EnsemblID | Class | Ref | Counted |
| --- | --- | --- | --- | --- | --- | --- | --- | --- | --- | --- | --- | --- | --- |
| rs806371:88856363:T:G | chr6:88855057-88857334(-) | Junction | -0.3598 | 6.03E-09 | 1.815E-06 | 0.6421 | rs806371 | chr6:88855057-88857334 | CNR1 | ENSG00000118432 | AltStartEnd | T | G |
| rs9359767:88839274:G:A | chr6:88855057-88857334(-) | Junction | -0.4274 | 1.629E-06 | 0.0002902 | 1 | rs9359767 | chr6:88855057-88857334 | CNR1 | ENSG00000118432 | AltStartEnd | G | A |
| rs9353524:88837616:C:G | chr6:88855057-88857334(-) | Junction | -0.2865 | 3.872E-06 | 0.0006242 | 1 | rs9353524 | chr6:88855057-88857334 | CNR1 | ENSG00000118432 | AltStartEnd | C | G |
| rs10944343:88831383:C:T | chr6:88855057-88857334(-) | Junction | 0.2193 | 6.098E-06 | 0.0009304 | 1 | rs10944343 | chr6:88855057-88857334 | CNR1 | ENSG00000118432 | AltStartEnd | C | T |
| rs2057403:88841558:G:C | chr6:88855057-88857334(-) | Junction | -0.3338 | 0.00001145 | 0.001612 | 1 | rs2057403 | chr6:88855057-88857334 | CNR1 | ENSG00000118432 | AltStartEnd | G | C |
| rs9353525 | chr6:88855057-88857334(-) | Junction | -0.3338 | 0.00001145 | 0.001612 | 1 | rs9353525 | chr6:88855057-88857334 | CNR1 | ENSG00000118432 | AltStartEnd | G | A |
| rs806370:88856331:C:T | chr6:88855057-88857334(-) | Junction | -0.3343 | 0.00001155 | 0.001623 | 1 | rs806370 | chr6:88855057-88857334 | CNR1 | ENSG00000118432 | AltStartEnd | C | T |
| rs10485171 | chr6:88855057-88857334(-) | Junction | 0.2083 | 0.00001762 | 0.002339 | 1 | rs10485171 | chr6:88855057-88857334 | CNR1 | ENSG00000118432 | AltStartEnd | A | G |
| rs806372:88856563:G:C | chr6:88855057-88857334(-) | Junction | -0.3386 | 0.00002912 | 0.003601 | 1 | rs806372 | chr6:88855057-88857334 | CNR1 | ENSG00000118432 | AltStartEnd | G | C |
| rs806366 | chr6:88855057-88857334(-) | Junction | 0.2208 | 0.00003972 | 0.00469 | 1 | rs806366 | chr6:88855057-88857334 | CNR1 | ENSG00000118432 | AltStartEnd | T | C |
| rs1078602 | chr6:88855057-88857334(-) | Junction | 0.1932 | 0.00007836 | 0.008315 | 1 | rs1078602 | chr6:88855057-88857334 | CNR1 | ENSG00000118432 | AltStartEnd | G | A |
| rs9450891:88830721:C:T | chr6:88855057-88857334(-) | Junction | 0.2177 | 7.591E-06 | 0.001126 | 1 | rs9450891 | chr6:88855057-88857334 | CNR1 | ENSG00000118432 | AltStartEnd | C | T |
| rs7751158 | chr6:88855057-88857334(-) | Junction | 0.1988 | 0.00006889 | 0.007466 | 1 | rs7751158 | chr6:88855057-88857334 | CNR1 | ENSG00000118432 | AltStartEnd | C | T |
| rs2146274:88827788:T:C | chr6:88855057-88857334(-) | Junction | 0.2022 | 0.0000431 | 0.005027 | 1 | rs2146274 | chr6:88855057-88857334 | CNR1 | ENSG00000118432 | AltStartEnd | T | C |
| rs200789989:89149356:C:CT | chr6:88857526-88860201(-) | Junction | 0.3405 | 4.227E-06 | 0.0006743 | 1 | rs565085193 | chr6:88857526-88860201 | CNR1 | ENSG00000118432 | AltStartEnd | C | CT |
| rs806371:88856363:T:G | chr6:88857526-88875521(-) | Junction | -0.2301 | 0.00006485 | 0.007097 | 1 | rs806371 | chr6:88857526-88875521 | CNR1 | ENSG00000118432 | ExonSkip | T | G |
| rs806368 | chr6:88855057-88857334(-) | Junction | -0.3438 | 7.29E-08 | 0.00001776 | 1 | rs806368 | chr6:88855057-88857334 | CNR1 | ENSG00000118432 | AltStartEnd | T | C |
| rs806375:88858521:A:T | chr6:88874014-88875521(-) | Junction | 0.1515 | 0.00009062 | 0.009388 | 1 | rs806375 | chr6:88874014-88875521 | CNR1 | ENSG00000118432 | InEns | A | T |
| rs7766029 | chr6:88874014-88875521(-) | Junction | -0.1533 | 0.00002934 | 0.003624 | 1 | rs7766029 | chr6:88874014-88875521 | CNR1 | ENSG00000118432 | InEns | C | T |
| 6:88629536:C:CGGGGA | e407416 | Exon | 0.09 | 7.591E-06 | 0.001126 | 1 | rs11397926 | chr6:88857383-88860882 | CNR1 | ENSG00000118432 | InEns | C | CGGGGA |
| rs6925605:88629973:C:T | e407416 | Exon | 0.08964 | 8.101E-06 | 0.001192 | 1 | rs6925605 | chr6:88857383-88860882 | CNR1 | ENSG00000118432 | InEns | C | T |
| rs806371:88856363:T:G | e407416 | Exon | -0.1002 | 0.00001026 | 0.001465 | 1 | rs806371 | chr6:88857383-88860882 | CNR1 | ENSG00000118432 | InEns | T | G |
| rs6910293 | e407416 | Exon | 0.08789 | 0.00001075 | 0.001525 | 1 | rs6910293 | chr6:88857383-88860882 | CNR1 | ENSG00000118432 | InEns | C | T |
| rs806370:88856331:C:T | e407416 | Exon | -0.1057 | 0.0001441 | 0.01377 | 1 | rs806370 | chr6:88857383-88860882 | CNR1 | ENSG00000118432 | InEns | C | T |
| rs9450891:88830721:C:T | e407416 | Exon | 0.07055 | 0.00007008 | 0.007574 | 1 | rs9450891 | chr6:88857383-88860882 | CNR1 | ENSG00000118432 | InEns | C | T |
| rs806375:88858521:A:T | e407416 | Exon | 0.07499 | 0.00007324 | 0.00786 | 1 | rs806375 | chr6:88857383-88860882 | CNR1 | ENSG00000118432 | InEns | A | T |
| rs113221376:88858839:C:T | e407416 | Exon | 0.09336 | 0.00007606 | 0.008111 | 1 | rs113221376 | chr6:88857383-88860882 | CNR1 | ENSG00000118432 | InEns | C | T |
| rs7766029 | e407418 | Exon | -0.1042 | 0.00004129 | 0.004847 | 1 | rs7766029 | chr6:88873976-88874062 | CNR1 | ENSG00000118432 | InEns | C | T |
| rs6918611:88639794:T:C | e407418 | Exon | 0.1323 | 0.00005464 | 0.006143 | 1 | rs6918611 | chr6:88873976-88874062 | CNR1 | ENSG00000118432 | InEns | T | C |
| rs6918611:88639794:T:C | e407430 | Exon | 0.1454 | 0.00003923 | 0.004641 | 1 | rs6918611 | chr6:88873976-88874013 | CNR1 | ENSG00000118432 | InEns | T | C |
| rs7766029 | e407430 | Exon | -0.108 | 0.00008263 | 0.008692 | 1 | rs7766029 | chr6:88873976-88874013 | CNR1 | ENSG00000118432 | InEns | C | T |
| rs806371:88856363:T:G | e407431 | Exon | -0.1631 | 5.45E-07 | 0.0001091 | 1 | rs806371 | chr6:88857383-88857525 | CNR1 | ENSG00000118432 | InEns | T | G |
| rs806375:88858521:A:T | e407431 | Exon | 0.1172 | 0.00001581 | 0.00213 | 1 | rs806375 | chr6:88857383-88857525 | CNR1 | ENSG00000118432 | InEns | A | T |
| rs806370:88856331:C:T | e407431 | Exon | -0.1395 | 0.0004977 | 0.03702 | 1 | rs806370 | chr6:88857383-88857525 | CNR1 | ENSG00000118432 | InEns | C | T |
| rs35951010:88844731:G:C | chr6:88857526-88875521(-) | Junction | 0.3093 | 0.00008069 | 0.008522 | 1 | rs35951010 | chr6:88857526-88875521 | CNR1 | ENSG00000118432 | ExonSkip | G | C |
