# Supplementary material for: Cannabinoid receptor CNR1 expression and DNA methylation in human prefrontal cortex, hippocampus and caudate in brain development and schizophrenia
Source: Transl Psychiatry. 2020 May 19;10:158. doi: 10.1038/s41398-020-0832-8 (PMC7237456; doi:10.1038/s41398-020-0832-8)
Supplement: Supplementary file 5 — Supplementary Table 4 [file 41398_2020_832_MOESM5_ESM.docx]

Supplementary Table 4. CNR1 5'UTR TFBIND Results

Part I. TFBIND results for the longest 5’UTR of CNR1

| TF Matrix ID | TF Label | Similarity | Position | Strandness | Consensus Sequence | Subsequence |
| --- | --- | --- | --- | --- | --- | --- |
| M00244 | V$NGFIC_01 | 0.7381 | 4 | (-) | WTGCGTGGGYGG | CCGGCGCCGCCT |
| M00255 | V$GC_01 | 0.849584 | 5 | (-) | NRGGGGCGGGGCNK | CGGCGCCGCCTCCC |
| M00002 | V$E47_01 | 0.776617 | 6 | (-) | NSNGCAGGTGKNCNN | GGCGCCGCCTCCCGC |
| M00196 | V$SP1_Q6 | 0.890847 | 6 | (-) | NGGGGGCGGGGYN | GGCGCCGCCTCCC |
| M00008 | V$SP1_01 | 0.828992 | 7 | (-) | GRGGCRGGGW | GCGCCGCCTC |
| M00115 | V$TAXCREB_02 | 0.679917 | 7 | (+) | RTGACGCATAYCCCC | GCGCCGCCTCCCGCA |
| M00196 | V$SP1_Q6 | 0.811152 | 9 | (-) | NGGGGGCGGGGYN | GCCGCCTCCCGCA |
| M00075 | V$GATA1_01 | 0.776407 | 10 | (-) | SNNGATNNNN | CCGCCTCCCG |
| M00189 | V$AP2_Q6 | 0.861443 | 11 | (+) | MKCCCSCNGGCG | CGCCTCCCGCAC |
| M00141 | V$LYF1_01 | 0.831587 | 13 | (-) | TTTGGGAGR | CCTCCCGCA |
| M00196 | V$SP1_Q6 | 0.762493 | 13 | (-) | NGGGGGCGGGGYN | CCTCCCGCACGCT |
| M00237 | V$AHRARNT_02 | 0.719894 | 13 | (-) | GRGKATYGCGTGMSWNSCC | CCTCCCGCACGCTACTCCC |
| M00244 | V$NGFIC_01 | 0.720078 | 13 | (-) | WTGCGTGGGYGG | CCTCCCGCACGC |
| M00189 | V$AP2_Q6 | 0.890165 | 14 | (+) | MKCCCSCNGGCG | CTCCCGCACGCT |
| M00189 | V$AP2_Q6 | 0.794347 | 14 | (-) | MKCCCSCNGGCG | CTCCCGCACGCT |
| M00055 | V$NMYC_01 | 0.752129 | 15 | (+) | NNNCACGTGNNN | TCCCGCACGCTA |
| M00236 | V$ARNT_01 | 0.836824 | 15 | (-) | NNNNNCACGTGNNNNN | TCCCGCACGCTACTCC |
| M00244 | V$NGFIC_01 | 0.722625 | 15 | (-) | WTGCGTGGGYGG | TCCCGCACGCTA |
| M00123 | V$MYCMAX_02 | 0.802732 | 17 | (+) | NANCACGTGNNW | CCGCACGCTACT |
| M00235 | V$AHRARNT_01 | 0.850419 | 17 | (-) | KNNKNNTYGCGTGCMS | CCGCACGCTACTCCCT |
| M00033 | V$P300_01 | 0.827198 | 21 | (-) | NNNRGGAGTNNNNS | ACGCTACTCCCTCT |
| M00255 | V$GC_01 | 0.907888 | 21 | (-) | NRGGGGCGGGGCNK | ACGCTACTCCCTCT |
| M00196 | V$SP1_Q6 | 0.820884 | 22 | (-) | NGGGGGCGGGGYN | CGCTACTCCCTCT |
| M00084 | V$MZF1_02 | 0.833042 | 25 | (-) | KNNNKAGGGGNAA | TACTCCCTCTGCC |
| M00189 | V$AP2_Q6 | 0.788603 | 27 | (+) | MKCCCSCNGGCG | CTCCCTCTGCCA |
| M00175 | V$AP4_Q5 | 0.781284 | 28 | (-) | NNCAGCTGNN | TCCCTCTGCC |
| M00176 | V$AP4_Q6 | 0.790028 | 28 | (-) | CWCAGCTGGN | TCCCTCTGCC |
| M00184 | V$MYOD_Q6 | 0.813078 | 28 | (+) | NNCANCTGNY | TCCCTCTGCC |
| M00023 | V$HOX13_01 | 0.753109 | 29 | (+) | TGCNNNNWYCCYCATTAKTNNNNNMNNYCN | CCCTCTGCCACCCCTTCCTTCTCCACTTCT |
| M00217 | V$USF_C | 0.803086 | 29 | (+) | NCACGTGN | CCCTCTGC |
| M00249 | V$CHOP_01 | 0.774374 | 31 | (+) | NNRTGCAATMCCC | CTCTGCCACCCCT |
| M00196 | V$SP1_Q6 | 0.756707 | 32 | (-) | NGGGGGCGGGGYN | TCTGCCACCCCTT |
| M00033 | V$P300_01 | 0.812883 | 33 | (-) | NNNRGGAGTNNNNS | CTGCCACCCCTTCC |
| M00008 | V$SP1_01 | 0.848528 | 34 | (-) | GRGGCRGGGW | TGCCACCCCT |
| M00008 | V$SP1_01 | 0.85957 | 35 | (-) | GRGGCRGGGW | GCCACCCCTT |
| M00244 | V$NGFIC_01 | 0.761606 | 36 | (-) | WTGCGTGGGYGG | CCACCCCTTCCT |
| M00253 | V$CAP_01 | 0.886151 | 36 | (+) | NCANNNNN | CCACCCCT |
| M00255 | V$GC_01 | 0.802303 | 36 | (-) | NRGGGGCGGGGCNK | CCACCCCTTCCTTC |
| M00084 | V$MZF1_02 | 0.823544 | 37 | (-) | KNNNKAGGGGNAA | CACCCCTTCCTTC |
| M00196 | V$SP1_Q6 | 0.760126 | 37 | (-) | NGGGGGCGGGGYN | CACCCCTTCCTTC |
| M00255 | V$GC_01 | 0.864527 | 37 | (-) | NRGGGGCGGGGCNK | CACCCCTTCCTTCT |
| M00196 | V$SP1_Q6 | 0.754077 | 38 | (-) | NGGGGGCGGGGYN | ACCCCTTCCTTCT |
| M00025 | V$ELK1_02 | 0.823998 | 39 | (-) | NNNNCCGGAARYNN | CCCCTTCCTTCTCC |
| M00097 | V$PAX6_01 | 0.753298 | 39 | (+) | NNNNTTCACGCWTSANTKNNN | CCCCTTCCTTCTCCACTTCTT |
| M00023 | V$HOX13_01 | 0.758489 | 40 | (+) | TGCNNNNWYCCYCATTAKTNNNNNMNNYCN | CCCTTCCTTCTCCACTTCTTTTCCGCCTCC |
| M00108 | V$NRF2_01 | 0.856589 | 40 | (-) | ACCGGAAGNS | CCCTTCCTTC |
| M00127 | V$GATA1_03 | 0.791524 | 43 | (-) | RNSNNGATAANNGN | TTCCTTCTCCACTT |
| M00187 | V$USF_Q6 | 0.800951 | 50 | (+) | GYCACGTGNC | TCCACTTCTT |
| M00253 | V$CAP_01 | 0.890586 | 51 | (+) | NCANNNNN | CCACTTCT |
| M00108 | V$NRF2_01 | 0.79845 | 52 | (-) | ACCGGAAGNS | CACTTCTTTT |
| M00144 | V$PAX5_02 | 0.78829 | 53 | (-) | RRMSWGANWYCTNRAGCGKRACSRYNSM | ACTTCTTTTCCGCCTCCGCCTCTTCTTG |
| M00127 | V$GATA1_03 | 0.849829 | 54 | (-) | RNSNNGATAANNGN | CTTCTTTTCCGCCT |
| M00025 | V$ELK1_02 | 0.814203 | 56 | (-) | NNNNCCGGAARYNN | TCTTTTCCGCCTCC |
| M00074 | V$CETS1P54_02 | 0.854712 | 56 | (-) | NNAMMGGAWRWNN | TCTTTTCCGCCTC |
| M00108 | V$NRF2_01 | 0.8125 | 57 | (-) | ACCGGAAGNS | CTTTTCCGCC |
| M00255 | V$GC_01 | 0.781725 | 57 | (-) | NRGGGGCGGGGCNK | CTTTTCCGCCTCCG |
| M00050 | V$E2F_02 | 0.847265 | 58 | (+) | TTTSGCGC | TTTTCCGC |
| M00196 | V$SP1_Q6 | 0.769332 | 58 | (-) | NGGGGGCGGGGYN | TTTTCCGCCTCCG |
| M00008 | V$SP1_01 | 0.853341 | 59 | (-) | GRGGCRGGGW | TTTCCGCCTC |
| M00189 | V$AP2_Q6 | 0.780331 | 59 | (+) | MKCCCSCNGGCG | TTTCCGCCTCCG |
| M00007 | V$ELK1_01 | 0.766867 | 60 | (-) | NNNACMGGAAGTNCNN | TTCCGCCTCCGCCTCT |
| M00084 | V$MZF1_02 | 0.87878 | 60 | (-) | KNNNKAGGGGNAA | TTCCGCCTCCGCC |
| M00243 | V$EGR1_01 | 0.89054 | 62 | (-) | WTGCGTGGGCGK | CCGCCTCCGCCT |
| M00244 | V$NGFIC_01 | 0.891871 | 62 | (-) | WTGCGTGGGYGG | CCGCCTCCGCCT |
| M00245 | V$EGR3_01 | 0.837292 | 62 | (-) | NTGCGTGGGCGK | CCGCCTCCGCCT |
| M00246 | V$EGR2_01 | 0.844875 | 62 | (-) | NTGCGTRGGCGK | CCGCCTCCGCCT |
| M00108 | V$NRF2_01 | 0.786822 | 63 | (-) | ACCGGAAGNS | CGCCTCCGCC |
| M00255 | V$GC_01 | 0.927976 | 63 | (-) | NRGGGGCGGGGCNK | CGCCTCCGCCTCTT |
| M00196 | V$SP1_Q6 | 0.876644 | 64 | (-) | NGGGGGCGGGGYN | GCCTCCGCCTCTT |
| M00008 | V$SP1_01 | 0.810872 | 65 | (-) | GRGGCRGGGW | CCTCCGCCTC |
| M00244 | V$NGFIC_01 | 0.748286 | 65 | (-) | WTGCGTGGGYGG | CCTCCGCCTCTT |
| M00084 | V$MZF1_02 | 0.825544 | 66 | (-) | KNNNKAGGGGNAA | CTCCGCCTCTTCT |
| M00244 | V$NGFIC_01 | 0.754554 | 68 | (-) | WTGCGTGGGYGG | CCGCCTCTTCTT |
| M00146 | V$HSF1_01 | 0.833399 | 69 | (-) | RGAANRTTCN | CGCCTCTTCT |
| M00147 | V$HSF2_01 | 0.83586 | 69 | (-) | NGAANNWTCK | CGCCTCTTCT |
| M00108 | V$NRF2_01 | 0.84593 | 72 | (-) | ACCGGAAGNS | CTCTTCTTGT |
| M00011 | V$EVI1_06 | 0.777076 | 73 | (-) | ACAAGATAA | TCTTCTTGT |
| M00079 | V$EVI1_02 | 0.809473 | 73 | (-) | AGAYAAGATAA | TCTTCTTGTCT |
| M00080 | V$EVI1_03 | 0.761111 | 73 | (-) | AGATAAGATAA | TCTTCTTGTCT |
| M00082 | V$EVI1_05 | 0.816805 | 73 | (-) | AGATAAGATAN | TCTTCTTGTCT |
| M00127 | V$GATA1_03 | 0.826801 | 74 | (-) | RNSNNGATAANNGN | CTTCTTGTCTCCCG |
| M00160 | V$SRY_02 | 0.780681 | 74 | (-) | NWWAACAAWANN | CTTCTTGTCTCC |
| M00180 | V$E2F_Q6 | 0.789953 | 78 | (-) | NNGCGCGAAANTK | TTGTCTCCCGCGG |
| M00008 | V$SP1_01 | 0.785108 | 79 | (-) | GRGGCRGGGW | TGTCTCCCGC |
| M00108 | V$NRF2_01 | 0.796027 | 80 | (-) | ACCGGAAGNS | GTCTCCCGCG |
| M00050 | V$E2F_02 | 0.828381 | 81 | (+) | TTTSGCGC | TCTCCCGC |
| M00187 | V$USF_Q6 | 0.795918 | 82 | (+) | GYCACGTGNC | CTCCCGCGGC |
| M00189 | V$AP2_Q6 | 0.876149 | 82 | (+) | MKCCCSCNGGCG | CTCCCGCGGCGC |
| M00175 | V$AP4_Q5 | 0.792165 | 83 | (-) | NNCAGCTGNN | TCCCGCGGCG |
| M00176 | V$AP4_Q6 | 0.816838 | 83 | (-) | CWCAGCTGGN | TCCCGCGGCG |
| M00017 | V$ATF_01 | 0.764228 | 85 | (-) | CNSTGACGTNNNYC | CCGCGGCGCCAGCG |
| M00007 | V$ELK1_01 | 0.757515 | 86 | (+) | NNNACMGGAAGTNCNN | CGCGGCGCCAGCGCCT |
| M00072 | V$CP2_01 | 0.786449 | 86 | (+) | GCNMNAMCMAG | CGCGGCGCCAG |
| M00177 | V$CREB_Q2 | 0.798744 | 86 | (-) | NSTGACGTAANN | CGCGGCGCCAGC |
| M00178 | V$CREB_Q4 | 0.800606 | 86 | (-) | NSTGACGTMANN | CGCGGCGCCAGC |
| M00179 | V$CREBP1_Q2 | 0.796811 | 86 | (-) | NSTGACGTMASN | CGCGGCGCCAGC |
| M00114 | V$TAXCREB_01 | 0.762222 | 87 | (-) | GGGGGTTGACGYANA | GCGGCGCCAGCGCCT |
| M00085 | V$ZID_01 | 0.849358 | 88 | (+) | NGGCTCYATCAYC | CGGCGCCAGCGCC |
| M00113 | V$CREB_02 | 0.771759 | 88 | (-) | NNGNTGACGYNN | CGGCGCCAGCGC |
| M00005 | V$AP4_01 | 0.779251 | 89 | (+) | WGARYCAGCTGYGGNCNK | GGCGCCAGCGCCTTCCCT |
| M00050 | V$E2F_02 | 0.743405 | 90 | (-) | TTTSGCGC | GCGCCAGC |
| M00075 | V$GATA1_01 | 0.804541 | 91 | (-) | SNNGATNNNN | CGCCAGCGCC |
| M00175 | V$AP4_Q5 | 0.790261 | 92 | (+) | NNCAGCTGNN | GCCAGCGCCT |
| M00176 | V$AP4_Q6 | 0.785517 | 92 | (+) | CWCAGCTGGN | GCCAGCGCCT |
| M00255 | V$GC_01 | 0.797648 | 94 | (-) | NRGGGGCGGGGCNK | CAGCGCCTTCCCTT |
| M00146 | V$HSF1_01 | 0.786136 | 95 | (-) | RGAANRTTCN | AGCGCCTTCC |
| M00147 | V$HSF2_01 | 0.821072 | 95 | (-) | NGAANNWTCK | AGCGCCTTCC |
| M00255 | V$GC_01 | 0.809897 | 95 | (-) | NRGGGGCGGGGCNK | AGCGCCTTCCCTTG |
| M00051 | V$NFKAPPAB50_01 | 0.770546 | 96 | (-) | GGGGATYCCC | GCGCCTTCCC |
| M00025 | V$ELK1_02 | 0.808081 | 97 | (-) | NNNNCCGGAARYNN | CGCCTTCCCTTGGC |
| M00086 | V$IK1_01 | 0.807959 | 97 | (-) | NNNTGGGAATRCC | CGCCTTCCCTTGG |
| M00087 | V$IK2_01 | 0.854712 | 98 | (-) | NNNYGGGAWNNN | GCCTTCCCTTGG |
| M00108 | V$NRF2_01 | 0.811773 | 98 | (-) | ACCGGAAGNS | GCCTTCCCTT |
| M00189 | V$AP2_Q6 | 0.797105 | 100 | (+) | MKCCCSCNGGCG | CTTCCCTTGGCC |
| M00227 | V$VMYB_02 | 0.831528 | 102 | (-) | NSYAACGGN | TCCCTTGGC |
| M00254 | V$CAAT_01 | 0.852866 | 103 | (-) | NNNRRCCAATSA | CCCTTGGCCCGG |
| M00193 | V$NF1_Q6 | 0.800429 | 104 | (+) | NNTTGGCNNNNNNCCNNN | CCTTGGCCCGGGCGGGGG |
| M00050 | V$E2F_02 | 0.743405 | 105 | (+) | TTTSGCGC | CTTGGCCC |
| M00072 | V$CP2_01 | 0.783019 | 106 | (-) | GCNMNAMCMAG | TTGGCCCGGGC |
| M00189 | V$AP2_Q6 | 0.813189 | 106 | (-) | MKCCCSCNGGCG | TTGGCCCGGGCG |
| M00243 | V$EGR1_01 | 0.778068 | 107 | (+) | WTGCGTGGGCGK | TGGCCCGGGCGG |
| M00244 | V$NGFIC_01 | 0.770617 | 107 | (+) | WTGCGTGGGYGG | TGGCCCGGGCGG |
| M00245 | V$EGR3_01 | 0.7425 | 107 | (+) | NTGCGTGGGCGK | TGGCCCGGGCGG |
| M00246 | V$EGR2_01 | 0.759419 | 107 | (+) | NTGCGTRGGCGK | TGGCCCGGGCGG |
| M00189 | V$AP2_Q6 | 0.853171 | 108 | (+) | MKCCCSCNGGCG | GGCCCGGGCGGG |
| M00189 | V$AP2_Q6 | 0.81296 | 110 | (-) | MKCCCSCNGGCG | CCCGGGCGGGGG |
| M00196 | V$SP1_Q6 | 0.889532 | 110 | (+) | NGGGGGCGGGGYN | CCCGGGCGGGGGC |
| M00255 | V$GC_01 | 0.803283 | 110 | (+) | NRGGGGCGGGGCNK | CCCGGGCGGGGGCC |
| M00196 | V$SP1_Q6 | 0.754077 | 111 | (+) | NGGGGGCGGGGYN | CCGGGCGGGGGCC |
| M00008 | V$SP1_01 | 0.838052 | 112 | (+) | GRGGCRGGGW | CGGGCGGGGG |
| M00189 | V$AP2_Q6 | 0.88534 | 112 | (-) | MKCCCSCNGGCG | CGGGCGGGGGCC |
| M00008 | V$SP1_01 | 0.800963 | 113 | (+) | GRGGCRGGGW | GGGCGGGGGC |
| M00115 | V$TAXCREB_02 | 0.62474 | 113 | (-) | RTGACGCATAYCCCC | GGGCGGGGGCCTCGG |
| M00189 | V$AP2_Q6 | 0.829044 | 113 | (-) | MKCCCSCNGGCG | GGGCGGGGGCCT |
| M00243 | V$EGR1_01 | 0.781884 | 113 | (+) | WTGCGTGGGCGK | GGGCGGGGGCCT |
| M00244 | V$NGFIC_01 | 0.775514 | 113 | (+) | WTGCGTGGGYGG | GGGCGGGGGCCT |
| M00245 | V$EGR3_01 | 0.767292 | 113 | (+) | NTGCGTGGGCGK | GGGCGGGGGCCT |
| M00246 | V$EGR2_01 | 0.756051 | 113 | (+) | NTGCGTRGGCGK | GGGCGGGGGCCT |
| M00083 | V$MZF1_01 | 0.846997 | 114 | (+) | NGNGGGGA | GGCGGGGG |
| M00196 | V$SP1_Q6 | 0.759074 | 116 | (+) | NGGGGGCGGGGYN | CGGGGGCCTCGGC |
| M00007 | V$ELK1_01 | 0.768871 | 117 | (-) | NNNACMGGAAGTNCNN | GGGGGCCTCGGCTCCC |
| M00037 | V$NFE2_01 | 0.803146 | 119 | (-) | TGCTGASTCAY | GGGCCTCGGCT |
| M00175 | V$AP4_Q5 | 0.792165 | 123 | (+) | NNCAGCTGNN | CTCGGCTCCC |
| M00176 | V$AP4_Q6 | 0.806815 | 123 | (+) | CWCAGCTGGN | CTCGGCTCCC |
| M00261 | V$OLF1_01 | 0.809188 | 123 | (+) | NNCNANTCCCYNGRGARNNKGN | CTCGGCTCCCTGCAGAGCTCTC |
| M00261 | V$OLF1_01 | 0.793526 | 123 | (-) | NNCNANTCCCYNGRGARNNKGN | CTCGGCTCCCTGCAGAGCTCTC |
| M00032 | V$CETS1P54_01 | 0.823194 | 126 | (-) | NCMGGAWGYN | GGCTCCCTGC |
| M00189 | V$AP2_Q6 | 0.78148 | 126 | (+) | MKCCCSCNGGCG | GGCTCCCTGCAG |
| M00189 | V$AP2_Q6 | 0.805377 | 128 | (+) | MKCCCSCNGGCG | CTCCCTGCAGAG |
| M00175 | V$AP4_Q5 | 0.821001 | 130 | (-) | NNCAGCTGNN | CCCTGCAGAG |
| M00253 | V$CAP_01 | 0.874322 | 134 | (+) | NCANNNNN | GCAGAGCT |
| M00175 | V$AP4_Q5 | 0.799238 | 135 | (+) | NNCAGCTGNN | CAGAGCTCTC |
| M00175 | V$AP4_Q5 | 0.806311 | 135 | (-) | NNCAGCTGNN | CAGAGCTCTC |
| M00176 | V$AP4_Q6 | 0.806815 | 135 | (+) | CWCAGCTGGN | CAGAGCTCTC |
| M00176 | V$AP4_Q6 | 0.780005 | 135 | (-) | CWCAGCTGGN | CAGAGCTCTC |
| M00147 | V$HSF2_01 | 0.801479 | 136 | (-) | NGAANNWTCK | AGAGCTCTCC |
| M00004 | V$CMYB_01 | 0.749837 | 143 | (+) | NCNRNNGRCNGTTGGKGG | TCCGTAGTCAGTGGGGGA |
| M00227 | V$VMYB_02 | 0.797533 | 143 | (-) | NSYAACGGN | TCCGTAGTC |
| M00172 | V$AP1FJ_Q2 | 0.88612 | 144 | (-) | RSTGACTNMNW | CCGTAGTCAGT |
| M00173 | V$AP1_Q2 | 0.909224 | 144 | (-) | RSTGACTNMNW | CCGTAGTCAGT |
| M00174 | V$AP1_Q6 | 0.875141 | 144 | (-) | NNTGACTCANN | CCGTAGTCAGT |
| M00188 | V$AP1_Q4 | 0.909729 | 144 | (-) | RSTGACTMANN | CCGTAGTCAGT |
| M00261 | V$OLF1_01 | 0.766641 | 144 | (-) | NNCNANTCCCYNGRGARNNKGN | CCGTAGTCAGTGGGGGATATTT |
| M00199 | V$AP1_C | 0.80379 | 145 | (+) | NTGASTCAG | CGTAGTCAG |
| M00199 | V$AP1_C | 0.78105 | 145 | (-) | NTGASTCAG | CGTAGTCAG |
| M00254 | V$CAAT_01 | 0.796385 | 145 | (+) | NNNRRCCAATSA | CGTAGTCAGTGG |
| M00185 | V$NFY_Q6 | 0.770298 | 147 | (+) | TRRCCAATSRN | TAGTCAGTGGG |
| M00175 | V$AP4_Q5 | 0.81012 | 149 | (+) | NNCAGCTGNN | GTCAGTGGGG |
| M00176 | V$AP4_Q6 | 0.806815 | 149 | (+) | CWCAGCTGGN | GTCAGTGGGG |
| M00183 | V$MYB_Q6 | 0.826141 | 149 | (-) | NNNAACKGNC | GTCAGTGGGG |
| M00185 | V$NFY_Q6 | 0.812693 | 149 | (-) | TRRCCAATSRN | GTCAGTGGGGG |
| M00084 | V$MZF1_02 | 0.83929 | 150 | (+) | KNNNKAGGGGNAA | TCAGTGGGGGATA |
| M00227 | V$VMYB_02 | 0.787605 | 150 | (-) | NSYAACGGN | TCAGTGGGG |
| M00253 | V$CAP_01 | 0.906851 | 150 | (+) | NCANNNNN | TCAGTGGG |
| M00083 | V$MZF1_01 | 0.897242 | 152 | (+) | NGNGGGGA | AGTGGGGG |
| M00008 | V$SP1_01 | 0.785957 | 153 | (+) | GRGGCRGGGW | GTGGGGGATA |
| M00083 | V$MZF1_01 | 0.920665 | 153 | (+) | NGNGGGGA | GTGGGGGA |
| M00115 | V$TAXCREB_02 | 0.68585 | 154 | (-) | RTGACGCATAYCCCC | TGGGGGATATTTCGT |
| M00008 | V$SP1_01 | 0.810872 | 155 | (+) | GRGGCRGGGW | GGGGGATATT |
| M00194 | V$NFKB_Q6 | 0.816335 | 155 | (+) | NGGGGAMTTTCCNN | GGGGGATATTTCGT |
| M00075 | V$GATA1_01 | 0.899803 | 156 | (+) | SNNGATNNNN | GGGGATATTT |
| M00076 | V$GATA2_01 | 0.931439 | 156 | (+) | NNNGATRNNN | GGGGATATTT |
| M00208 | V$NFKB_C | 0.800837 | 156 | (+) | NGGGACTTTCCA | GGGGATATTTCG |
| M00052 | V$NFKAPPAB65_01 | 0.804551 | 157 | (+) | GGGRATTTCC | GGGATATTTC |
| M00053 | V$CREL_01 | 0.89238 | 157 | (+) | SGGRNWTTCC | GGGATATTTC |
| M00054 | V$NFKAPPAB_01 | 0.853933 | 157 | (+) | GGGAMTTYCC | GGGATATTTC |
| M00053 | V$CREL_01 | 0.826752 | 158 | (-) | SGGRNWTTCC | GGATATTTCG |
| M00146 | V$HSF1_01 | 0.840095 | 158 | (+) | RGAANRTTCN | GGATATTTCG |
| M00146 | V$HSF1_01 | 0.76605 | 158 | (-) | RGAANRTTCN | GGATATTTCG |
| M00147 | V$HSF2_01 | 0.881701 | 158 | (+) | NGAANNWTCK | GGATATTTCG |
| M00147 | V$HSF2_01 | 0.867652 | 158 | (-) | NGAANNWTCK | GGATATTTCG |
| M00162 | V$OCT1_06 | 0.808203 | 158 | (+) | CWNAWTKWSATRYN | GGATATTTCGTTCT |
| M00249 | V$CHOP_01 | 0.809628 | 158 | (-) | NNRTGCAATMCCC | GGATATTTCGTTC |
| M00180 | V$E2F_Q6 | 0.768744 | 160 | (-) | NNGCGCGAAANTK | ATATTTCGTTCTA |
| M00050 | V$E2F_02 | 0.743405 | 163 | (+) | TTTSGCGC | TTTCGTTC |
| M00072 | V$CP2_01 | 0.787307 | 164 | (-) | GCNMNAMCMAG | TTCGTTCTAGC |
| M00189 | V$AP2_Q6 | 0.790441 | 167 | (+) | MKCCCSCNGGCG | GTTCTAGCGGAC |
| M00141 | V$LYF1_01 | 0.844365 | 169 | (+) | TTTGGGAGR | TCTAGCGGA |
| M00227 | V$VMYB_02 | 0.854994 | 169 | (+) | NSYAACGGN | TCTAGCGGA |
| M00209 | V$NFY_C | 0.765428 | 172 | (-) | NCTGATTGGYTASY | AGCGGACAACCAGC |
| M00007 | V$ELK1_01 | 0.786907 | 173 | (-) | NNNACMGGAAGTNCNN | GCGGACAACCAGCCCC |
| M00002 | V$E47_01 | 0.774608 | 174 | (-) | NSNGCAGGTGKNCNN | CGGACAACCAGCCCC |
| M00004 | V$CMYB_01 | 0.757858 | 174 | (-) | NCNRNNGRCNGTTGGKGG | CGGACAACCAGCCCCTGA |
| M00183 | V$MYB_Q6 | 0.854357 | 176 | (+) | NNNAACKGNC | GACAACCAGC |
| M00005 | V$AP4_01 | 0.797839 | 177 | (+) | WGARYCAGCTGYGGNCNK | ACAACCAGCCCCTGAGCT |
| M00255 | V$GC_01 | 0.83415 | 177 | (-) | NRGGGGCGGGGCNK | ACAACCAGCCCCTG |
| M00196 | V$SP1_Q6 | 0.800105 | 178 | (-) | NGGGGGCGGGGYN | CAACCAGCCCCTG |
| M00272 | V$P53_02 | 0.822978 | 178 | (+) | NGRCWTGYCY | CAACCAGCCC |
| M00008 | V$SP1_01 | 0.91761 | 179 | (-) | GRGGCRGGGW | AACCAGCCCC |
| M00075 | V$GATA1_01 | 0.773939 | 179 | (-) | SNNGATNNNN | AACCAGCCCC |
| M00008 | V$SP1_01 | 0.794734 | 180 | (-) | GRGGCRGGGW | ACCAGCCCCT |
| M00155 | V$ARP1_01 | 0.819543 | 181 | (+) | TGARCCYTTGAMCCYW | CCAGCCCCTGAGCTGG |
| M00253 | V$CAP_01 | 0.897486 | 181 | (+) | NCANNNNN | CCAGCCCC |
| M00001 | V$MYOD_01 | 0.861614 | 182 | (-) | SRACAGGTGKYG | CAGCCCCTGAGC |
| M00277 | V$LMO2COM_01 | 0.81965 | 182 | (-) | SNNCAGGTGNNN | CAGCCCCTGAGC |
| M00005 | V$AP4_01 | 0.792219 | 183 | (-) | WGARYCAGCTGYGGNCNK | AGCCCCTGAGCTGGGCGA |
| M00175 | V$AP4_Q5 | 0.803047 | 183 | (-) | NNCAGCTGNN | AGCCCCTGAG |
| M00176 | V$AP4_Q6 | 0.823603 | 183 | (-) | CWCAGCTGGN | AGCCCCTGAG |
| M00189 | V$AP2_Q6 | 0.784697 | 183 | (+) | MKCCCSCNGGCG | AGCCCCTGAGCT |
| M00189 | V$AP2_Q6 | 0.781939 | 183 | (-) | MKCCCSCNGGCG | AGCCCCTGAGCT |
| M00217 | V$USF_C | 0.817175 | 184 | (+) | NCACGTGN | GCCCCTGA |
| M00139 | V$AHR_01 | 0.766773 | 185 | (+) | CYYCNRRSTNGCGTGASW | CCCCTGAGCTGGGCGAGA |
| M00072 | V$CP2_01 | 0.805746 | 188 | (-) | GCNMNAMCMAG | CTGAGCTGGGC |
| M00175 | V$AP4_Q5 | 0.863439 | 188 | (+) | NNCAGCTGNN | CTGAGCTGGG |
| M00175 | V$AP4_Q5 | 0.832699 | 188 | (-) | NNCAGCTGNN | CTGAGCTGGG |
| M00176 | V$AP4_Q6 | 0.867452 | 188 | (+) | CWCAGCTGGN | CTGAGCTGGG |
| M00189 | V$AP2_Q6 | 0.845358 | 188 | (-) | MKCCCSCNGGCG | CTGAGCTGGGCG |
| M00242 | V$PPARA_01 | 0.770594 | 188 | (+) | CWRAWCTAGGNCAAAGGTCA | CTGAGCTGGGCGAGAGGTGC |
| M00273 | V$R_01 | 0.737631 | 188 | (+) | NNGKCCNCSNRNYGTGGTGYN | CTGAGCTGGGCGAGAGGTGCC |
| M00237 | V$AHRARNT_02 | 0.742916 | 190 | (+) | GRGKATYGCGTGMSWNSCC | GAGCTGGGCGAGAGGTGCC |
| M00141 | V$LYF1_01 | 0.828009 | 192 | (+) | TTTGGGAGR | GCTGGGCGA |
| M00196 | V$SP1_Q6 | 0.756707 | 192 | (+) | NGGGGGCGGGGYN | GCTGGGCGAGAGG |
| M00050 | V$E2F_02 | 0.743405 | 195 | (-) | TTTSGCGC | GGGCGAGA |
| M00193 | V$NF1_Q6 | 0.819406 | 195 | (-) | NNTTGGCNNNNNNCCNNN | GGGCGAGAGGTGCCAAGG |
| M00075 | V$GATA1_01 | 0.805528 | 196 | (+) | SNNGATNNNN | GGCGAGAGGT |
| M00115 | V$TAXCREB_02 | 0.638831 | 196 | (-) | RTGACGCATAYCCCC | GGCGAGAGGTGCCAA |
| M00001 | V$MYOD_01 | 0.866929 | 198 | (+) | SRACAGGTGKYG | CGAGAGGTGCCA |
| M00277 | V$LMO2COM_01 | 0.800269 | 198 | (+) | SNNCAGGTGNNN | CGAGAGGTGCCA |
| M00175 | V$AP4_Q5 | 0.788357 | 199 | (-) | NNCAGCTGNN | GAGAGGTGCC |
| M00176 | V$AP4_Q6 | 0.763217 | 199 | (-) | CWCAGCTGGN | GAGAGGTGCC |
| M00184 | V$MYOD_Q6 | 0.842381 | 199 | (-) | NNCANCTGNY | GAGAGGTGCC |
| M00261 | V$OLF1_01 | 0.843644 | 199 | (+) | NNCNANTCCCYNGRGARNNKGN | GAGAGGTGCCAAGGGAGCTTCT |
| M00261 | V$OLF1_01 | 0.774471 | 199 | (-) | NNCNANTCCCYNGRGARNNKGN | GAGAGGTGCCAAGGGAGCTTCT |
| M00281 | V$RFX1_02 | 0.791694 | 201 | (+) | NNGTNRCNATRGYAACNNN | GAGGTGCCAAGGGAGCTTC |
| M00155 | V$ARP1_01 | 0.819689 | 202 | (-) | TGARCCYTTGAMCCYW | AGGTGCCAAGGGAGCT |
| M00050 | V$E2F_02 | 0.743405 | 204 | (-) | TTTSGCGC | GTGCCAAG |
| M00189 | V$AP2_Q6 | 0.782169 | 204 | (+) | MKCCCSCNGGCG | GTGCCAAGGGAG |
| M00187 | V$USF_Q6 | 0.810176 | 206 | (-) | GYCACGTGNC | GCCAAGGGAG |
| M00227 | V$VMYB_02 | 0.831528 | 206 | (+) | NSYAACGGN | GCCAAGGGA |
| M00255 | V$GC_01 | 0.808672 | 208 | (+) | NRGGGGCGGGGCNK | CAAGGGAGCTTCTG |
| M00192 | V$GR_Q6 | 0.791295 | 209 | (+) | NNNNNNCNNTNTGTNCTNN | AAGGGAGCTTCTGTCCCGA |
| M00194 | V$NFKB_Q6 | 0.788557 | 209 | (+) | NGGGGAMTTTCCNN | AAGGGAGCTTCTGT |
| M00007 | V$ELK1_01 | 0.768871 | 211 | (-) | NNNACMGGAAGTNCNN | GGGAGCTTCTGTCCCG |
| M00054 | V$NFKAPPAB_01 | 0.787765 | 211 | (+) | GGGAMTTYCC | GGGAGCTTCT |
| M00065 | V$TAL1BETAE47_01 | 0.776106 | 211 | (-) | NNNAACAGATGKTNNN | GGGAGCTTCTGTCCCG |
| M00066 | V$TAL1ALPHAE47_01 | 0.779996 | 211 | (-) | NNNAACAGATGKTNNN | GGGAGCTTCTGTCCCG |
| M00070 | V$TAL1BETAITF2_01 | 0.798577 | 211 | (-) | NNNAACAGATGKTNNN | GGGAGCTTCTGTCCCG |
| M00146 | V$HSF1_01 | 0.776684 | 211 | (-) | RGAANRTTCN | GGGAGCTTCT |
| M00147 | V$HSF2_01 | 0.880222 | 211 | (+) | NGAANNWTCK | GGGAGCTTCT |
| M00176 | V$AP4_Q6 | 0.763217 | 211 | (-) | CWCAGCTGGN | GGGAGCTTCT |
| M00205 | V$GRE_C | 0.77913 | 211 | (+) | GGTACAANNTGTYCTK | GGGAGCTTCTGTCCCG |
| M00155 | V$ARP1_01 | 0.795405 | 212 | (+) | TGARCCYTTGAMCCYW | GGAGCTTCTGTCCCGA |
| M00057 | V$COMP1_01 | 0.772934 | 213 | (+) | NNTNWKGATTGRCNRSRANMRRNN | GAGCTTCTGTCCCGAGGACCAGGG |
| M00175 | V$AP4_Q5 | 0.781284 | 214 | (-) | NNCAGCTGNN | AGCTTCTGTC |
| M00184 | V$MYOD_Q6 | 0.832202 | 214 | (+) | NNCANCTGNY | AGCTTCTGTC |
| M00087 | V$IK2_01 | 0.861911 | 218 | (-) | NNNYGGGAWNNN | TCTGTCCCGAGG |
| M00189 | V$AP2_Q6 | 0.795956 | 220 | (+) | MKCCCSCNGGCG | TGTCCCGAGGAC |
| M00189 | V$AP2_Q6 | 0.804917 | 220 | (-) | MKCCCSCNGGCG | TGTCCCGAGGAC |
| M00189 | V$AP2_Q6 | 0.877987 | 221 | (+) | MKCCCSCNGGCG | GTCCCGAGGACC |
| M00189 | V$AP2_Q6 | 0.800322 | 221 | (-) | MKCCCSCNGGCG | GTCCCGAGGACC |
| M00121 | V$USF_01 | 0.744327 | 228 | (+) | NNRYCACGTGRYNN | GGACCAGGGGATGC |
| M00121 | V$USF_01 | 0.744327 | 228 | (-) | NNRYCACGTGRYNN | GGACCAGGGGATGC |
| M00122 | V$USF_02 | 0.782163 | 228 | (+) | NNRNCACGTGNYNN | GGACCAGGGGATGC |
| M00122 | V$USF_02 | 0.782163 | 228 | (-) | NNRNCACGTGNYNN | GGACCAGGGGATGC |
| M00162 | V$OCT1_06 | 0.803125 | 229 | (+) | CWNAWTKWSATRYN | GACCAGGGGATGCG |
| M00189 | V$AP2_Q6 | 0.788143 | 229 | (-) | MKCCCSCNGGCG | GACCAGGGGATG |
| M00220 | V$SREBP1_01 | 0.779159 | 230 | (-) | NATCACGTGAY | ACCAGGGGATG |
| M00074 | V$CETS1P54_02 | 0.832461 | 231 | (+) | NNAMMGGAWRWNN | CCAGGGGATGCGA |
| M00083 | V$MZF1_01 | 0.863241 | 231 | (+) | NGNGGGGA | CCAGGGGA |
| M00217 | V$USF_C | 0.82053 | 231 | (-) | NCACGTGN | CCAGGGGA |
| M00072 | V$CP2_01 | 0.786021 | 232 | (-) | GCNMNAMCMAG | CAGGGGATGCG |
| M00115 | V$TAXCREB_02 | 0.628004 | 232 | (-) | RTGACGCATAYCCCC | CAGGGGATGCGAAGG |
| M00075 | V$GATA1_01 | 0.897828 | 234 | (+) | SNNGATNNNN | GGGGATGCGA |
| M00076 | V$GATA2_01 | 0.890843 | 234 | (+) | NNNGATRNNN | GGGGATGCGA |
| M00115 | V$TAXCREB_02 | 0.631415 | 234 | (-) | RTGACGCATAYCCCC | GGGGATGCGAAGGAT |
| M00195 | V$OCT1_Q6 | 0.825873 | 234 | (+) | NNNNATGCAAATNAN | GGGGATGCGAAGGAT |
| M00210 | V$OCT_C | 0.772213 | 236 | (-) | CTNATTTGCATAY | GGATGCGAAGGAT |
| M00243 | V$EGR1_01 | 0.761398 | 238 | (+) | WTGCGTGGGCGK | ATGCGAAGGATG |
| M00244 | V$NGFIC_01 | 0.765524 | 238 | (+) | WTGCGTGGGYGG | ATGCGAAGGATG |
| M00246 | V$EGR2_01 | 0.754368 | 238 | (+) | NTGCGTRGGCGK | ATGCGAAGGATG |
| M00007 | V$ELK1_01 | 0.773547 | 239 | (+) | NNNACMGGAAGTNCNN | TGCGAAGGATGTCTCC |
| M00025 | V$ELK1_02 | 0.823079 | 239 | (+) | NNNNCCGGAARYNN | TGCGAAGGATGTCT |
| M00074 | V$CETS1P54_02 | 0.858966 | 240 | (+) | NNAMMGGAWRWNN | GCGAAGGATGTCT |
| M00104 | V$CDPCR1_01 | 0.79225 | 240 | (-) | NATCGATCGS | GCGAAGGATG |
| M00115 | V$TAXCREB_02 | 0.648769 | 240 | (+) | RTGACGCATAYCCCC | GCGAAGGATGTCTCC |
| M00032 | V$CETS1P54_01 | 0.875611 | 242 | (+) | NCMGGAWGYN | GAAGGATGTC |
| M00106 | V$CDPCR3HD_01 | 0.854671 | 242 | (+) | NATYGATSSS | GAAGGATGTC |
| M00194 | V$NFKB_Q6 | 0.789594 | 242 | (-) | NGGGGAMTTTCCNN | GAAGGATGTCTCCC |
| M00075 | V$GATA1_01 | 0.806515 | 243 | (+) | SNNGATNNNN | AAGGATGTCT |
| M00076 | V$GATA2_01 | 0.866937 | 243 | (+) | NNNGATRNNN | AAGGATGTCT |
| M00272 | V$P53_02 | 0.821219 | 243 | (+) | NGRCWTGYCY | AAGGATGTCT |
| M00272 | V$P53_02 | 0.841149 | 243 | (-) | NGRCWTGYCY | AAGGATGTCT |
| M00023 | V$HOX13_01 | 0.732066 | 245 | (-) | TGCNNNNWYCCYCATTAKTNNNNNMNNYCN | GGATGTCTCCCACTCCAGGCGAGGAGCGCT |
| M00147 | V$HSF2_01 | 0.791867 | 245 | (+) | NGAANNWTCK | GGATGTCTCC |
| M00147 | V$HSF2_01 | 0.816266 | 245 | (-) | NGAANNWTCK | GGATGTCTCC |
| M00115 | V$TAXCREB_02 | 0.68585 | 247 | (+) | RTGACGCATAYCCCC | ATGTCTCCCACTCCA |
| M00008 | V$SP1_01 | 0.785108 | 248 | (-) | GRGGCRGGGW | TGTCTCCCAC |
| M00244 | V$NGFIC_01 | 0.802155 | 250 | (-) | WTGCGTGGGYGG | TCTCCCACTCCA |
| M00246 | V$EGR2_01 | 0.792044 | 250 | (-) | NTGCGTRGGCGK | TCTCCCACTCCA |
| M00033 | V$P300_01 | 0.91411 | 251 | (-) | NNNRGGAGTNNNNS | CTCCCACTCCAGGC |
| M00189 | V$AP2_Q6 | 0.8233 | 251 | (+) | MKCCCSCNGGCG | CTCCCACTCCAG |
| M00072 | V$CP2_01 | 0.786878 | 252 | (+) | GCNMNAMCMAG | TCCCACTCCAG |
| M00139 | V$AHR_01 | 0.776011 | 252 | (-) | CYYCNRRSTNGCGTGASW | TCCCACTCCAGGCGAGGA |
| M00189 | V$AP2_Q6 | 0.783088 | 257 | (+) | MKCCCSCNGGCG | CTCCAGGCGAGG |
| M00073 | V$DELTAEF1_01 | 0.810916 | 258 | (-) | NNNCACCTNAN | TCCAGGCGAGG |
| M00008 | V$SP1_01 | 0.795866 | 260 | (+) | GRGGCRGGGW | CAGGCGAGGA |
| M00196 | V$SP1_Q6 | 0.769069 | 260 | (+) | NGGGGGCGGGGYN | CAGGCGAGGAGCG |
| M00255 | V$GC_01 | 0.794953 | 260 | (+) | NRGGGGCGGGGCNK | CAGGCGAGGAGCGC |
| M00084 | V$MZF1_02 | 0.813547 | 261 | (+) | KNNNKAGGGGNAA | AGGCGAGGAGCGC |
| M00083 | V$MZF1_01 | 0.846997 | 262 | (+) | NGNGGGGA | GGCGAGGA |
| M00115 | V$TAXCREB_02 | 0.611391 | 262 | (-) | RTGACGCATAYCCCC | GGCGAGGAGCGCTCT |
| M00069 | V$YY1_02 | 0.782578 | 266 | (-) | NNNCGGCCATCTTGNCTSNW | AGGAGCGCTCTTGGCTGAGG |
| M00146 | V$HSF1_01 | 0.765262 | 267 | (+) | RGAANRTTCN | GGAGCGCTCT |
| M00146 | V$HSF1_01 | 0.774714 | 267 | (-) | RGAANRTTCN | GGAGCGCTCT |
| M00147 | V$HSF2_01 | 0.815527 | 267 | (+) | NGAANNWTCK | GGAGCGCTCT |
| M00147 | V$HSF2_01 | 0.791497 | 267 | (-) | NGAANNWTCK | GGAGCGCTCT |
| M00185 | V$NFY_Q6 | 0.792909 | 272 | (-) | TRRCCAATSRN | GCTCTTGGCTG |
| M00254 | V$CAAT_01 | 0.859362 | 273 | (-) | NNNRRCCAATSA | CTCTTGGCTGAG |
| M00057 | V$COMP1_01 | 0.797006 | 274 | (-) | NNTNWKGATTGRCNRSRANMRRNN | TCTTGGCTGAGGGCAAAGAGATTG |
| M00193 | V$NF1_Q6 | 0.90052 | 274 | (+) | NNTTGGCNNNNNNCCNNN | TCTTGGCTGAGGGCAAAG |
| M00072 | V$CP2_01 | 0.856775 | 275 | (-) | GCNMNAMCMAG | CTTGGCTGAGG |
| M00114 | V$TAXCREB_01 | 0.727879 | 275 | (+) | GGGGGTTGACGYANA | CTTGGCTGAGGGCAA |
| M00193 | V$NF1_Q6 | 0.794307 | 275 | (-) | NNTTGGCNNNNNNCCNNN | CTTGGCTGAGGGCAAAGA |
| M00200 | V$CAAT_C | 0.736608 | 275 | (-) | ACCAATCANCNNGCYYSNCNCWNNT | CTTGGCTGAGGGCAAAGAGATTGAA |
| M00084 | V$MZF1_02 | 0.865784 | 277 | (+) | KNNNKAGGGGNAA | TGGCTGAGGGCAA |
| M00113 | V$CREB_02 | 0.770444 | 277 | (+) | NNGNTGACGYNN | TGGCTGAGGGCA |
| M00084 | V$MZF1_02 | 0.820545 | 278 | (+) | KNNNKAGGGGNAA | GGCTGAGGGCAAA |
| M00239 | V$T3R_01 | 0.793299 | 278 | (+) | SNNTRAGGTCACGSNN | GGCTGAGGGCAAAGAG |
| M00039 | V$CREB_01 | 0.775797 | 281 | (+) | TGACGTMA | TGAGGGCA |
| M00039 | V$CREB_01 | 0.774627 | 281 | (-) | TGACGTMA | TGAGGGCA |
| M00057 | V$COMP1_01 | 0.803952 | 281 | (+) | NNTNWKGATTGRCNRSRANMRRNN | TGAGGGCAAAGAGATTGAAAGAGG |
| M00155 | V$ARP1_01 | 0.778683 | 283 | (-) | TGARCCYTTGAMCCYW | AGGGCAAAGAGATTGA |
| M00075 | V$GATA1_01 | 0.787759 | 290 | (+) | SNNGATNNNN | AGAGATTGAA |
| M00077 | V$GATA3_01 | 0.890563 | 291 | (+) | NNGATARNG | GAGATTGAA |
| M00059 | V$YY1_01 | 0.778178 | 294 | (-) | NNNNNCCATNTWNNNWN | ATTGAAAGAGGGGAGTC |
| M00238 | V$BARBIE_01 | 0.818226 | 294 | (+) | ATNNAAAGCNGRNGG | ATTGAAAGAGGGGAG |
| M00084 | V$MZF1_02 | 0.820545 | 297 | (+) | KNNNKAGGGGNAA | GAAAGAGGGGAGT |
| M00115 | V$TAXCREB_02 | 0.657223 | 298 | (-) | RTGACGCATAYCCCC | AAAGAGGGGAGTCAC |
| M00083 | V$MZF1_01 | 0.960332 | 300 | (+) | NGNGGGGA | AGAGGGGA |
| M00033 | V$P300_01 | 0.837764 | 301 | (+) | NNNRGGAGTNNNNS | GAGGGGAGTCACAT |
| M00194 | V$NFKB_Q6 | 0.838101 | 301 | (-) | NGGGGAMTTTCCNN | GAGGGGAGTCACAT |
| M00008 | V$SP1_01 | 0.821348 | 302 | (+) | GRGGCRGGGW | AGGGGAGTCA |
| M00037 | V$NFE2_01 | 0.792797 | 302 | (+) | TGCTGASTCAY | AGGGGAGTCAC |
| M00178 | V$CREB_Q4 | 0.746845 | 302 | (-) | NSTGACGTMANN | AGGGGAGTCACA |
| M00208 | V$NFKB_C | 0.806067 | 302 | (-) | NGGGACTTTCCA | AGGGGAGTCACA |
| M00051 | V$NFKAPPAB50_01 | 0.862728 | 303 | (+) | GGGGATYCCC | GGGGAGTCAC |
| M00051 | V$NFKAPPAB50_01 | 0.787206 | 303 | (-) | GGGGATYCCC | GGGGAGTCAC |
| M00054 | V$NFKAPPAB_01 | 0.866916 | 303 | (+) | GGGAMTTYCC | GGGGAGTCAC |
| M00054 | V$NFKAPPAB_01 | 0.803246 | 303 | (-) | GGGAMTTYCC | GGGGAGTCAC |
| M00114 | V$TAXCREB_01 | 0.72 | 303 | (+) | GGGGGTTGACGYANA | GGGGAGTCACATAAT |
| M00115 | V$TAXCREB_02 | 0.634678 | 303 | (-) | RTGACGCATAYCCCC | GGGGAGTCACATAAT |
| M00172 | V$AP1FJ_Q2 | 0.874448 | 303 | (-) | RSTGACTNMNW | GGGGAGTCACA |
| M00174 | V$AP1_Q6 | 0.886047 | 303 | (-) | NNTGACTCANN | GGGGAGTCACA |
| M00188 | V$AP1_Q4 | 0.858798 | 303 | (-) | RSTGACTMANN | GGGGAGTCACA |
| M00237 | V$AHRARNT_02 | 0.783058 | 303 | (-) | GRGKATYGCGTGMSWNSCC | GGGGAGTCACATAATCAGC |
| M00199 | V$AP1_C | 0.808746 | 304 | (+) | NTGASTCAG | GGGAGTCAC |
| M00199 | V$AP1_C | 0.801166 | 304 | (-) | NTGASTCAG | GGGAGTCAC |
| M00162 | V$OCT1_06 | 0.808203 | 305 | (-) | CWNAWTKWSATRYN | GGAGTCACATAATC |
| M00121 | V$USF_01 | 0.751715 | 306 | (+) | NNRYCACGTGRYNN | GAGTCACATAATCA |
| M00121 | V$USF_01 | 0.751715 | 306 | (-) | NNRYCACGTGRYNN | GAGTCACATAATCA |
| M00122 | V$USF_02 | 0.788378 | 306 | (+) | NNRNCACGTGNYNN | GAGTCACATAATCA |
| M00122 | V$USF_02 | 0.788378 | 306 | (-) | NNRNCACGTGNYNN | GAGTCACATAATCA |
| M00206 | V$HNF1_C | 0.817031 | 306 | (-) | NGTTAATKAWTNACCAM | GAGTCACATAATCAGCA |
| M00035 | V$VMAF_01 | 0.803228 | 307 | (-) | NNNTGCTGACTCAGCANNN | AGTCACATAATCAGCATAC |
| M00045 | V$E4BP4_01 | 0.794592 | 307 | (+) | NRTTAYGTAAYN | AGTCACATAATC |
| M00045 | V$E4BP4_01 | 0.857828 | 307 | (-) | NRTTAYGTAAYN | AGTCACATAATC |
| M00177 | V$CREB_Q2 | 0.7741 | 307 | (+) | NSTGACGTAANN | AGTCACATAATC |
| M00178 | V$CREB_Q4 | 0.783443 | 307 | (+) | NSTGACGTMANN | AGTCACATAATC |
| M00220 | V$SREBP1_01 | 0.76573 | 307 | (+) | NATCACGTGAY | AGTCACATAAT |
| M00099 | V$S8_01 | 0.775474 | 308 | (+) | WNNANYYAATTANYNN | GTCACATAATCAGCAT |
| M00187 | V$USF_Q6 | 0.80123 | 308 | (+) | GYCACGTGNC | GTCACATAAT |
| M00220 | V$SREBP1_01 | 0.823427 | 308 | (-) | NATCACGTGAY | GTCACATAATC |
| M00228 | V$VBP_01 | 0.877001 | 308 | (+) | GTTACRTMAK | GTCACATAAT |
| M00260 | V$HLF_01 | 0.879285 | 308 | (+) | RTTACRYAAT | GTCACATAAT |
| M00260 | V$HLF_01 | 0.859005 | 308 | (-) | RTTACRYAAT | GTCACATAAT |
| M00040 | V$CREBP1_01 | 0.816013 | 309 | (+) | TTACGTAA | TCACATAA |
| M00040 | V$CREBP1_01 | 0.751577 | 309 | (-) | TTACGTAA | TCACATAA |
| M00113 | V$CREB_02 | 0.777544 | 309 | (-) | NNGNTGACGYNN | TCACATAATCAG |
| M00138 | V$OCT1_04 | 0.843162 | 309 | (-) | NNNNNNNWATGCAAATNNNWNNW | TCACATAATCAGCATACGTTTAT |
| M00099 | V$S8_01 | 0.782554 | 310 | (-) | WNNANYYAATTANYNN | CACATAATCAGCATAC |
| M00127 | V$GATA1_03 | 0.808182 | 310 | (-) | RNSNNGATAANNGN | CACATAATCAGCAT |
| M00137 | V$OCT1_03 | 0.907942 | 310 | (+) | NNNRTAATNANNN | CACATAATCAGCA |
| M00129 | V$HFH1_01 | 0.815939 | 311 | (-) | NAWTGTTTATWT | ACATAATCAGCA |
| M00135 | V$OCT1_01 | 0.785387 | 311 | (-) | NNNNWTATGCAAATNTNNN | ACATAATCAGCATACGTTT |
| M00172 | V$AP1FJ_Q2 | 0.816719 | 311 | (-) | RSTGACTNMNW | ACATAATCAGC |
| M00173 | V$AP1_Q2 | 0.853587 | 311 | (-) | RSTGACTNMNW | ACATAATCAGC |
| M00174 | V$AP1_Q6 | 0.791651 | 311 | (-) | NNTGACTCANN | ACATAATCAGC |
| M00188 | V$AP1_Q4 | 0.831961 | 311 | (-) | RSTGACTMANN | ACATAATCAGC |
| M00075 | V$GATA1_01 | 0.886476 | 312 | (-) | SNNGATNNNN | CATAATCAGC |
| M00076 | V$GATA2_01 | 0.839423 | 312 | (-) | NNNGATRNNN | CATAATCAGC |
| M00077 | V$GATA3_01 | 0.870625 | 312 | (-) | NNGATARNG | CATAATCAG |
| M00241 | V$NKX25_02 | 0.834741 | 312 | (+) | CWTAATTG | CATAATCA |
| M00161 | V$OCT1_05 | 0.874813 | 313 | (+) | MKNATTTGCATAYY | ATAATCAGCATACG |
| M00210 | V$OCT_C | 0.837036 | 313 | (+) | CTNATTTGCATAY | ATAATCAGCATAC |
| M00248 | V$OCT1_07 | 0.857325 | 315 | (-) | TNTATGNTAATT | AATCAGCATACG |
| M00122 | V$USF_02 | 0.760099 | 317 | (+) | NNRNCACGTGNYNN | TCAGCATACGTTTA |
| M00122 | V$USF_02 | 0.760099 | 317 | (-) | NNRNCACGTGNYNN | TCAGCATACGTTTA |
| M00253 | V$CAP_01 | 0.910301 | 317 | (+) | NCANNNNN | TCAGCATA |
| M00076 | V$GATA2_01 | 0.827695 | 318 | (+) | NNNGATRNNN | CAGCATACGT |
| M00123 | V$MYCMAX_02 | 0.829525 | 318 | (+) | NANCACGTGNNW | CAGCATACGTTT |
| M00253 | V$CAP_01 | 0.897979 | 320 | (+) | NCANNNNN | GCATACGT |
| M00003 | V$VMYB_01 | 0.782482 | 322 | (-) | AAYAACGGNN | ATACGTTTAT |
| M00137 | V$OCT1_03 | 0.851837 | 322 | (-) | NNNRTAATNANNN | ATACGTTTATGAA |
| M00138 | V$OCT1_04 | 0.795901 | 322 | (+) | NNNNNNNWATGCAAATNNNWNNW | ATACGTTTATGAAGATTACCCCC |
| M00099 | V$S8_01 | 0.761315 | 324 | (-) | WNNANYYAATTANYNN | ACGTTTATGAAGATTA |
| M00127 | V$GATA1_03 | 0.786379 | 324 | (-) | RNSNNGATAANNGN | ACGTTTATGAAGAT |
| M00135 | V$OCT1_01 | 0.73636 | 324 | (+) | NNNNWTATGCAAATNTNNN | ACGTTTATGAAGATTACCC |
| M00216 | V$TATA_C | 0.741484 | 325 | (+) | NCTATAAAAR | CGTTTATGAA |
| M00100 | V$CDXA_01 | 0.929868 | 326 | (+) | MTTTATR | GTTTATG |
| M00195 | V$OCT1_Q6 | 0.803493 | 326 | (+) | NNNNATGCAAATNAN | GTTTATGAAGATTAC |
| M00206 | V$HNF1_C | 0.804804 | 326 | (-) | NGTTAATKAWTNACCAM | GTTTATGAAGATTACCC |
| M00136 | V$OCT1_02 | 0.796968 | 327 | (-) | NNGAATATKCANNNN | TTTATGAAGATTACC |
| M00141 | V$LYF1_01 | 0.821365 | 327 | (+) | TTTGGGAGR | TTTATGAAG |
| M00248 | V$OCT1_07 | 0.794785 | 327 | (+) | TNTATGNTAATT | TTTATGAAGATT |
| M00017 | V$ATF_01 | 0.746132 | 328 | (+) | CNSTGACGTNNNYC | TTATGAAGATTACC |
| M00210 | V$OCT_C | 0.797254 | 328 | (-) | CTNATTTGCATAY | TTATGAAGATTAC |
| M00115 | V$TAXCREB_02 | 0.794423 | 330 | (+) | RTGACGCATAYCCCC | ATGAAGATTACCCCC |
| M00127 | V$GATA1_03 | 0.8317 | 330 | (+) | RNSNNGATAANNGN | ATGAAGATTACCCC |
| M00137 | V$OCT1_03 | 0.845516 | 331 | (-) | NNNRTAATNANNN | TGAAGATTACCCC |
| M00208 | V$NFKB_C | 0.764017 | 331 | (-) | NGGGACTTTCCA | TGAAGATTACCC |
| M00075 | V$GATA1_01 | 0.818855 | 332 | (+) | SNNGATNNNN | GAAGATTACC |
| M00076 | V$GATA2_01 | 0.844835 | 332 | (+) | NNNGATRNNN | GAAGATTACC |
| M00127 | V$GATA1_03 | 0.782215 | 333 | (-) | RNSNNGATAANNGN | AAGATTACCCCCAC |
| M00116 | V$CEBPA_01 | 0.851779 | 334 | (+) | NNATTRCNNAANNN | AGATTACCCCCACA |
| M00147 | V$HSF2_01 | 0.794085 | 334 | (-) | NGAANNWTCK | AGATTACCCC |
| M00255 | V$GC_01 | 0.797648 | 334 | (-) | NRGGGGCGGGGCNK | AGATTACCCCCACA |
| M00221 | V$SREBP1_02 | 0.720902 | 335 | (+) | KATCACCCCAC | GATTACCCCCA |
| M00008 | V$SP1_01 | 0.79983 | 336 | (-) | GRGGCRGGGW | ATTACCCCCA |
| M00008 | V$SP1_01 | 0.804077 | 337 | (-) | GRGGCRGGGW | TTACCCCCAC |
| M00084 | V$MZF1_02 | 0.858285 | 337 | (-) | KNNNKAGGGGNAA | TTACCCCCACAGC |
| M00004 | V$CMYB_01 | 0.752872 | 339 | (-) | NCNRNNGRCNGTTGGKGG | ACCCCCACAGCTGTGTGG |
| M00005 | V$AP4_01 | 0.817435 | 339 | (-) | WGARYCAGCTGYGGNCNK | ACCCCCACAGCTGTGTGG |
| M00083 | V$MZF1_01 | 0.86173 | 340 | (-) | NGNGGGGA | CCCCCACA |
| M00002 | V$E47_01 | 0.872037 | 341 | (-) | NSNGCAGGTGKNCNN | CCCCACAGCTGTGTG |
| M00066 | V$TAL1ALPHAE47_01 | 0.768018 | 341 | (+) | NNNAACAGATGKTNNN | CCCCACAGCTGTGTGG |
| M00066 | V$TAL1ALPHAE47_01 | 0.774112 | 341 | (-) | NNNAACAGATGKTNNN | CCCCACAGCTGTGTGG |
| M00071 | V$E47_02 | 0.815784 | 341 | (+) | NNNMRCAGGTGTTMNN | CCCCACAGCTGTGTGG |
| M00071 | V$E47_02 | 0.819842 | 341 | (-) | NNNMRCAGGTGTTMNN | CCCCACAGCTGTGTGG |
| M00139 | V$AHR_01 | 0.751779 | 341 | (+) | CYYCNRRSTNGCGTGASW | CCCCACAGCTGTGTGGCA |
| M00002 | V$E47_01 | 0.889715 | 342 | (+) | NSNGCAGGTGKNCNN | CCCACAGCTGTGTGG |
| M00105 | V$CDPCR3_01 | 0.756434 | 342 | (+) | CACCRATANNTATNG | CCCACAGCTGTGTGG |
| M00187 | V$USF_Q6 | 0.795639 | 342 | (+) | GYCACGTGNC | CCCACAGCTG |
| M00001 | V$MYOD_01 | 0.858071 | 343 | (+) | SRACAGGTGKYG | CCACAGCTGTGT |
| M00001 | V$MYOD_01 | 0.865945 | 343 | (-) | SRACAGGTGKYG | CCACAGCTGTGT |
| M00277 | V$LMO2COM_01 | 0.896904 | 343 | (+) | SNNCAGGTGNNN | CCACAGCTGTGT |
| M00277 | V$LMO2COM_01 | 0.875908 | 343 | (-) | SNNCAGGTGNNN | CCACAGCTGTGT |
| M00175 | V$AP4_Q5 | 0.967356 | 344 | (+) | NNCAGCTGNN | CACAGCTGTG |
| M00175 | V$AP4_Q5 | 0.967356 | 344 | (-) | NNCAGCTGNN | CACAGCTGTG |
| M00176 | V$AP4_Q6 | 0.956402 | 344 | (+) | CWCAGCTGGN | CACAGCTGTG |
| M00176 | V$AP4_Q6 | 0.956402 | 344 | (-) | CWCAGCTGGN | CACAGCTGTG |
| M00184 | V$MYOD_Q6 | 0.803516 | 344 | (+) | NNCANCTGNY | CACAGCTGTG |
| M00184 | V$MYOD_Q6 | 0.803516 | 344 | (-) | NNCANCTGNY | CACAGCTGTG |
| M00222 | V$TH1E47_01 | 0.795092 | 345 | (+) | NNNNGNRTCTGGMWTT | ACAGCTGTGTGGCAAG |
| M00253 | V$CAP_01 | 0.885165 | 345 | (+) | NCANNNNN | ACAGCTGT |
| M00201 | V$CEBP_C | 0.880429 | 348 | (+) | NGWNTKNKGYAAKNSAYA | GCTGTGTGGCAAGTGATC |
| M00209 | V$NFY_C | 0.792394 | 348 | (+) | NCTGATTGGYTASY | GCTGTGTGGCAAGT |
| M00109 | V$CEBPB_01 | 0.862308 | 349 | (+) | RNRTKNNGMAAKNN | CTGTGTGGCAAGTG |
| M00116 | V$CEBPA_01 | 0.805241 | 349 | (-) | NNATTRCNNAANNN | CTGTGTGGCAAGTG |
| M00057 | V$COMP1_01 | 0.793533 | 350 | (+) | NNTNWKGATTGRCNRSRANMRRNN | TGTGTGGCAAGTGATCAAAAAGGA |
| M00159 | V$CEBP_01 | 0.900486 | 350 | (+) | NNTKTGGWNANNN | TGTGTGGCAAGTG |
| M00192 | V$GR_Q6 | 0.775112 | 350 | (+) | NNNNNNCNNTNTGTNCTNN | TGTGTGGCAAGTGATCAAA |
| M00254 | V$CAAT_01 | 0.781135 | 350 | (-) | NNNRRCCAATSA | TGTGTGGCAAGT |
| M00251 | V$XBP1_01 | 0.752991 | 351 | (-) | NNGNTGACGTGKNNNWT | GTGTGGCAAGTGATCAA |
| M00236 | V$ARNT_01 | 0.782969 | 352 | (+) | NNNNNCACGTGNNNNN | TGTGGCAAGTGATCAA |
| M00236 | V$ARNT_01 | 0.787342 | 352 | (-) | NNNNNCACGTGNNNNN | TGTGGCAAGTGATCAA |
| M00254 | V$CAAT_01 | 0.796668 | 352 | (+) | NNNRRCCAATSA | TGTGGCAAGTGA |
| M00271 | V$AML1_01 | 0.897865 | 352 | (+) | TGTGGT | TGTGGC |
| M00118 | V$MYCMAX_01 | 0.73468 | 353 | (+) | NNACCACGTGGTNN | GTGGCAAGTGATCA |
| M00118 | V$MYCMAX_01 | 0.73468 | 353 | (-) | NNACCACGTGGTNN | GTGGCAAGTGATCA |
| M00119 | V$MAX_01 | 0.780194 | 353 | (+) | NNANCACGTGNTNN | GTGGCAAGTGATCA |
| M00119 | V$MAX_01 | 0.780194 | 353 | (-) | NNANCACGTGNTNN | GTGGCAAGTGATCA |
| M00121 | V$USF_01 | 0.794459 | 353 | (+) | NNRYCACGTGRYNN | GTGGCAAGTGATCA |
| M00121 | V$USF_01 | 0.794459 | 353 | (-) | NNRYCACGTGRYNN | GTGGCAAGTGATCA |
| M00122 | V$USF_02 | 0.836855 | 353 | (+) | NNRNCACGTGNYNN | GTGGCAAGTGATCA |
| M00122 | V$USF_02 | 0.836855 | 353 | (-) | NNRNCACGTGNYNN | GTGGCAAGTGATCA |
| M00055 | V$NMYC_01 | 0.751217 | 354 | (+) | NNNCACGTGNNN | TGGCAAGTGATC |
| M00055 | V$NMYC_01 | 0.770377 | 354 | (-) | NNNCACGTGNNN | TGGCAAGTGATC |
| M00123 | V$MYCMAX_02 | 0.818755 | 354 | (-) | NANCACGTGNNW | TGGCAAGTGATC |
| M00235 | V$AHRARNT_01 | 0.76857 | 354 | (-) | KNNKNNTYGCGTGCMS | TGGCAAGTGATCAAAA |
| M00272 | V$P53_02 | 0.844666 | 354 | (-) | NGRCWTGYCY | TGGCAAGTGA |
| M00184 | V$MYOD_Q6 | 0.80475 | 355 | (+) | NNCANCTGNY | GGCAAGTGAT |
| M00184 | V$MYOD_Q6 | 0.867983 | 355 | (-) | NNCANCTGNY | GGCAAGTGAT |
| M00187 | V$USF_Q6 | 0.868325 | 355 | (-) | GYCACGTGNC | GGCAAGTGAT |
| M00220 | V$SREBP1_01 | 0.846307 | 355 | (-) | NATCACGTGAY | GGCAAGTGATC |
| M00104 | V$CDPCR1_01 | 0.800716 | 356 | (-) | NATCGATCGS | GCAAGTGATC |
| M00113 | V$CREB_02 | 0.782277 | 356 | (-) | NNGNTGACGYNN | GCAAGTGATCAA |
| M00217 | V$USF_C | 0.840322 | 356 | (-) | NCACGTGN | GCAAGTGA |
| M00240 | V$NKX25_01 | 0.950176 | 356 | (+) | TYAAGTG | GCAAGTG |
| M00172 | V$AP1FJ_Q2 | 0.823344 | 359 | (+) | RSTGACTNMNW | AGTGATCAAAA |
| M00173 | V$AP1_Q2 | 0.815813 | 359 | (+) | RSTGACTNMNW | AGTGATCAAAA |
| M00188 | V$AP1_Q4 | 0.818847 | 359 | (+) | RSTGACTMANN | AGTGATCAAAA |
| M00098 | V$PAX2_01 | 0.753206 | 360 | (-) | NNNNGTCANGNRTKANNNN | GTGATCAAAAAGGAACAGG |
| M00160 | V$SRY_02 | 0.854668 | 360 | (+) | NWWAACAAWANN | GTGATCAAAAAG |
| M00280 | V$RFX1_01 | 0.815443 | 361 | (+) | NNGTNRCNWRGYAACNN | TGATCAAAAAGGAACAG |
| M00059 | V$YY1_01 | 0.812582 | 362 | (-) | NNNNNCCATNTWNNNWN | GATCAAAAAGGAACAGG |
| M00074 | V$CETS1P54_02 | 0.860602 | 366 | (+) | NNAMMGGAWRWNN | AAAAAGGAACAGG |
| M00056 | V$MYOGNF1_01 | 0.744126 | 367 | (-) | CRSCTGTNNNNTTTGGCACNSNGCCARNN | AAAAGGAACAGGACCAGAGAAGAGCAGGA |
| M00108 | V$NRF2_01 | 0.80814 | 368 | (+) | ACCGGAAGNS | AAAGGAACAG |
| M00078 | V$EVI1_01 | 0.730608 | 372 | (+) | WGAYAAGATAAGATAA | GAACAGGACCAGAGAA |
| M00032 | V$CETS1P54_01 | 0.829984 | 374 | (+) | NCMGGAWGYN | ACAGGACCAG |
| M00254 | V$CAAT_01 | 0.800904 | 375 | (+) | NNNRRCCAATSA | CAGGACCAGAGA |
| M00152 | V$SRF_01 | 0.726752 | 376 | (-) | ATGCCCATATATGGWNNT | AGGACCAGAGAAGAGCAG |
| M00078 | V$EVI1_01 | 0.781948 | 377 | (+) | WGAYAAGATAAGATAA | GGACCAGAGAAGAGCA |
| M00079 | V$EVI1_02 | 0.77939 | 377 | (+) | AGAYAAGATAA | GGACCAGAGAA |
| M00127 | V$GATA1_03 | 0.820676 | 378 | (+) | RNSNNGATAANNGN | GACCAGAGAAGAGC |
| M00011 | V$EVI1_06 | 0.777076 | 379 | (+) | ACAAGATAA | ACCAGAGAA |
| M00128 | V$GATA1_04 | 0.834865 | 379 | (+) | NNCWGATARNNNN | ACCAGAGAAGAGC |
| M00200 | V$CAAT_C | 0.757984 | 379 | (-) | ACCAATCANCNNGCYYSNCNCWNNT | ACCAGAGAAGAGCAGGAAAACTGGT |
| M00271 | V$AML1_01 | 0.873587 | 379 | (-) | TGTGGT | ACCAGA |
| M00078 | V$EVI1_01 | 0.724888 | 382 | (+) | WGAYAAGATAAGATAA | AGAGAAGAGCAGGAAA |
| M00079 | V$EVI1_02 | 0.770215 | 382 | (+) | AGAYAAGATAA | AGAGAAGAGCA |
| M00080 | V$EVI1_03 | 0.717284 | 382 | (+) | AGATAAGATAA | AGAGAAGAGCA |
| M00205 | V$GRE_C | 0.804585 | 383 | (-) | GGTACAANNTGTYCTK | GAGAAGAGCAGGAAAA |
| M00175 | V$AP4_Q5 | 0.781284 | 386 | (+) | NNCAGCTGNN | AAGAGCAGGA |
| M00024 | V$E2F_01 | 0.81968 | 387 | (+) | TWSGCGCGAAAAYKR | AGAGCAGGAAAACTG |
| M00025 | V$ELK1_02 | 0.816039 | 387 | (+) | NNNNCCGGAARYNN | AGAGCAGGAAAACT |
| M00074 | V$CETS1P54_02 | 0.862565 | 388 | (+) | NNAMMGGAWRWNN | GAGCAGGAAAACT |
| M00086 | V$IK1_01 | 0.818643 | 388 | (+) | NNNTGGGAATRCC | GAGCAGGAAAACT |
| M00087 | V$IK2_01 | 0.884817 | 388 | (+) | NNNYGGGAWNNN | GAGCAGGAAAAC |
| M00088 | V$IK3_01 | 0.816354 | 388 | (+) | TNYTGGGAATACC | GAGCAGGAAAACT |
| M00180 | V$E2F_Q6 | 0.819721 | 388 | (+) | NNGCGCGAAANTK | GAGCAGGAAAACT |
| M00127 | V$GATA1_03 | 0.844194 | 389 | (+) | RNSNNGATAANNGN | AGCAGGAAAACTGG |
| M00032 | V$CETS1P54_01 | 0.87887 | 390 | (+) | NCMGGAWGYN | GCAGGAAAAC |
| M00050 | V$E2F_02 | 0.828381 | 390 | (-) | TTTSGCGC | GCAGGAAA |
| M00108 | V$NRF2_01 | 0.791667 | 390 | (+) | ACCGGAAGNS | GCAGGAAAAC |
| M00181 | V$E2_Q6 | 0.753244 | 390 | (+) | NNACCRNNANCGGTRN | GCAGGAAAACTGGTCA |
| M00059 | V$YY1_01 | 0.856488 | 391 | (-) | NNNNNCCATNTWNNNWN | CAGGAAAACTGGTCAGA |
| M00003 | V$VMYB_01 | 0.806076 | 394 | (+) | AAYAACGGNN | GAAAACTGGT |
| M00156 | V$RORA1_01 | 0.777052 | 394 | (+) | NWAWNNAGGTCAN | GAAAACTGGTCAG |
| M00183 | V$MYB_Q6 | 0.895851 | 394 | (+) | NNNAACKGNC | GAAAACTGGT |
| M00185 | V$NFY_Q6 | 0.812179 | 395 | (-) | TRRCCAATSRN | AAAACTGGTCA |
| M00272 | V$P53_02 | 0.82034 | 396 | (+) | NGRCWTGYCY | AAACTGGTCA |
| M00272 | V$P53_02 | 0.860199 | 396 | (-) | NGRCWTGYCY | AAACTGGTCA |
| M00174 | V$AP1_Q6 | 0.767582 | 397 | (-) | NNTGACTCANN | AACTGGTCAGA |
| M00032 | V$CETS1P54_01 | 0.828897 | 403 | (+) | NCMGGAWGYN | TCAGAAAGCA |
| M00238 | V$BARBIE_01 | 0.782556 | 403 | (+) | ATNNAAAGCNGRNGG | TCAGAAAGCAGGCGC |
| M00100 | V$CDXA_01 | 0.919568 | 404 | (-) | MTTTATR | CAGAAAG |
| M00175 | V$AP4_Q5 | 0.781284 | 406 | (+) | NNCAGCTGNN | GAAAGCAGGC |
| M00222 | V$TH1E47_01 | 0.820824 | 406 | (-) | NNNNGNRTCTGGMWTT | GAAAGCAGGCGCCCTA |
| M00122 | V$USF_02 | 0.804537 | 407 | (+) | NNRNCACGTGNYNN | AAAGCAGGCGCCCT |
| M00122 | V$USF_02 | 0.804537 | 407 | (-) | NNRNCACGTGNYNN | AAAGCAGGCGCCCT |
| M00143 | V$PAX5_01 | 0.77079 | 408 | (-) | NCNNNRNKCANNGNWGNRKRGCSRSNNN | AAGCAGGCGCCCTAACCCTGGATTGCCC |
| M00272 | V$P53_02 | 0.814771 | 408 | (-) | NGRCWTGYCY | AAGCAGGCGC |
| M00184 | V$MYOD_Q6 | 0.812461 | 409 | (-) | NNCANCTGNY | AGCAGGCGCC |
| M00191 | V$ER_Q6 | 0.749762 | 409 | (+) | NNARGNNANNNTGACCYNN | AGCAGGCGCCCTAACCCTG |
| M00050 | V$E2F_02 | 0.743405 | 414 | (-) | TTTSGCGC | GCGCCCTA |
| M00072 | V$CP2_01 | 0.79331 | 416 | (+) | GCNMNAMCMAG | GCCCTAACCCT |
| M00098 | V$PAX2_01 | 0.767282 | 416 | (-) | NNNNGTCANGNRTKANNNN | GCCCTAACCCTGGATTGCC |
| M00076 | V$GATA2_01 | 0.780785 | 417 | (-) | NNNGATRNNN | CCCTAACCCT |
| M00104 | V$CDPCR1_01 | 0.791924 | 424 | (+) | NATCGATCGS | CCTGGATTGC |
| M00072 | V$CP2_01 | 0.806175 | 425 | (-) | GCNMNAMCMAG | CTGGATTGCCC |
| M00075 | V$GATA1_01 | 0.847976 | 425 | (+) | SNNGATNNNN | CTGGATTGCC |
| M00076 | V$GATA2_01 | 0.823636 | 425 | (+) | NNNGATRNNN | CTGGATTGCC |
| M00052 | V$NFKAPPAB65_01 | 0.793772 | 426 | (+) | GGGRATTTCC | TGGATTGCCC |
| M00054 | V$NFKAPPAB_01 | 0.780524 | 426 | (+) | GGGAMTTYCC | TGGATTGCCC |
| M00208 | V$NFKB_C | 0.759205 | 426 | (-) | NGGGACTTTCCA | TGGATTGCCCCC |
| M00272 | V$P53_02 | 0.815944 | 426 | (+) | NGRCWTGYCY | TGGATTGCCC |
| M00052 | V$NFKAPPAB65_01 | 0.803114 | 427 | (-) | GGGRATTTCC | GGATTGCCCC |
| M00053 | V$CREL_01 | 0.902948 | 427 | (-) | SGGRNWTTCC | GGATTGCCCC |
| M00054 | V$NFKAPPAB_01 | 0.859675 | 427 | (-) | GGGAMTTYCC | GGATTGCCCC |
| M00116 | V$CEBPA_01 | 0.800939 | 427 | (+) | NNATTRCNNAANNN | GGATTGCCCCCTGT |
| M00002 | V$E47_01 | 0.787465 | 429 | (-) | NSNGCAGGTGKNCNN | ATTGCCCCCTGTGGG |
| M00005 | V$AP4_01 | 0.787464 | 429 | (-) | WGARYCAGCTGYGGNCNK | ATTGCCCCCTGTGGGTCA |
| M00001 | V$MYOD_01 | 0.866929 | 431 | (-) | SRACAGGTGKYG | TGCCCCCTGTGG |
| M00277 | V$LMO2COM_01 | 0.783042 | 431 | (-) | SNNCAGGTGNNN | TGCCCCCTGTGG |
| M00175 | V$AP4_Q5 | 0.792165 | 432 | (-) | NNCAGCTGNN | GCCCCCTGTG |
| M00176 | V$AP4_Q6 | 0.833626 | 432 | (-) | CWCAGCTGGN | GCCCCCTGTG |
| M00187 | V$USF_Q6 | 0.7948 | 432 | (-) | GYCACGTGNC | GCCCCCTGTG |
| M00217 | V$USF_C | 0.86481 | 433 | (+) | NCACGTGN | CCCCCTGT |
| M00187 | V$USF_Q6 | 0.871121 | 434 | (-) | GYCACGTGNC | CCCCTGTGGG |
| M00189 | V$AP2_Q6 | 0.891774 | 434 | (-) | MKCCCSCNGGCG | CCCCTGTGGGTC |
| M00156 | V$RORA1_01 | 0.783103 | 435 | (+) | NWAWNNAGGTCAN | CCCTGTGGGTCAC |
| M00217 | V$USF_C | 0.828246 | 435 | (+) | NCACGTGN | CCCTGTGG |
| M00177 | V$CREB_Q2 | 0.75308 | 437 | (-) | NSTGACGTAANN | CTGTGGGTCACT |
| M00172 | V$AP1FJ_Q2 | 0.915142 | 438 | (-) | RSTGACTNMNW | TGTGGGTCACT |
| M00173 | V$AP1_Q2 | 0.899268 | 438 | (-) | RSTGACTNMNW | TGTGGGTCACT |
| M00174 | V$AP1_Q6 | 0.873261 | 438 | (-) | NNTGACTCANN | TGTGGGTCACT |
| M00188 | V$AP1_Q4 | 0.907289 | 438 | (-) | RSTGACTMANN | TGTGGGTCACT |
| M00191 | V$ER_Q6 | 0.7923 | 439 | (-) | NNARGNNANNNTGACCYNN | GTGGGTCACTTTCTCAGTC |
| M00199 | V$AP1_C | 0.810787 | 439 | (+) | NTGASTCAG | GTGGGTCAC |
| M00199 | V$AP1_C | 0.801166 | 439 | (-) | NTGASTCAG | GTGGGTCAC |
| M00113 | V$CREB_02 | 0.780962 | 440 | (+) | NNGNTGACGYNN | TGGGTCACTTTC |
| M00236 | V$ARNT_01 | 0.756041 | 440 | (-) | NNNNNCACGTGNNNNN | TGGGTCACTTTCTCAG |
| M00033 | V$P300_01 | 0.811861 | 441 | (-) | NNNRGGAGTNNNNS | GGGTCACTTTCTCA |
| M00007 | V$ELK1_01 | 0.780227 | 442 | (-) | NNNACMGGAAGTNCNN | GGTCACTTTCTCAGTC |
| M00187 | V$USF_Q6 | 0.835337 | 443 | (+) | GYCACGTGNC | GTCACTTTCT |
| M00253 | V$CAP_01 | 0.953179 | 444 | (+) | NCANNNNN | TCACTTTC |
| M00035 | V$VMAF_01 | 0.776257 | 446 | (-) | NNNTGCTGACTCAGCANNN | ACTTTCTCAGTCATTTTGA |
| M00173 | V$AP1_Q2 | 0.802928 | 446 | (-) | RSTGACTNMNW | ACTTTCTCAGT |
| M00174 | V$AP1_Q6 | 0.788642 | 446 | (-) | NNTGACTCANN | ACTTTCTCAGT |
| M00199 | V$AP1_C | 0.859475 | 447 | (+) | NTGASTCAG | CTTTCTCAG |
| M00199 | V$AP1_C | 0.78863 | 447 | (-) | NTGASTCAG | CTTTCTCAG |
| M00056 | V$MYOGNF1_01 | 0.790256 | 448 | (+) | CRSCTGTNNNNTTTGGCACNSNGCCARNN | TTTCTCAGTCATTTTGAGCTCAGCCTAAT |
| M00037 | V$NFE2_01 | 0.81391 | 449 | (+) | TGCTGASTCAY | TTCTCAGTCAT |
| M00172 | V$AP1FJ_Q2 | 0.915142 | 450 | (-) | RSTGACTNMNW | TCTCAGTCATT |
| M00173 | V$AP1_Q2 | 0.944949 | 450 | (-) | RSTGACTNMNW | TCTCAGTCATT |
| M00174 | V$AP1_Q6 | 0.896202 | 450 | (-) | NNTGACTCANN | TCTCAGTCATT |
| M00188 | V$AP1_Q4 | 0.926807 | 450 | (-) | RSTGACTMANN | TCTCAGTCATT |
| M00191 | V$ER_Q6 | 0.808935 | 451 | (+) | NNARGNNANNNTGACCYNN | CTCAGTCATTTTGAGCTCA |
| M00199 | V$AP1_C | 0.822449 | 451 | (+) | NTGASTCAG | CTCAGTCAT |
| M00199 | V$AP1_C | 0.861516 | 451 | (-) | NTGASTCAG | CTCAGTCAT |
| M00253 | V$CAP_01 | 0.95515 | 452 | (+) | NCANNNNN | TCAGTCAT |
| M00133 | V$TST1_01 | 0.879969 | 454 | (+) | NNKGAWTWANANTNN | AGTCATTTTGAGCTC |
| M00253 | V$CAP_01 | 0.935929 | 456 | (+) | NCANNNNN | TCATTTTG |
| M00160 | V$SRY_02 | 0.760423 | 457 | (-) | NWWAACAAWANN | CATTTTGAGCTC |
| M00239 | V$T3R_01 | 0.77986 | 457 | (-) | SNNTRAGGTCACGSNN | CATTTTGAGCTCAGCC |
| M00156 | V$RORA1_01 | 0.782619 | 458 | (+) | NWAWNNAGGTCAN | ATTTTGAGCTCAG |
| M00050 | V$E2F_02 | 0.762288 | 459 | (+) | TTTSGCGC | TTTTGAGC |
| M00175 | V$AP4_Q5 | 0.792165 | 461 | (-) | NNCAGCTGNN | TTGAGCTCAG |
| M00039 | V$CREB_01 | 0.774627 | 462 | (+) | TGACGTMA | TGAGCTCA |
| M00039 | V$CREB_01 | 0.774627 | 462 | (-) | TGACGTMA | TGAGCTCA |
| M00255 | V$GC_01 | 0.844194 | 463 | (-) | NRGGGGCGGGGCNK | GAGCTCAGCCTAAT |
| M00008 | V$SP1_01 | 0.834655 | 465 | (-) | GRGGCRGGGW | GCTCAGCCTA |
| M00084 | V$MZF1_02 | 0.820545 | 466 | (-) | KNNNKAGGGGNAA | CTCAGCCTAATCA |
| M00250 | V$GFI1_01 | 0.757683 | 466 | (+) | NNNNNNNAAATCASWGYNNNNNNN | CTCAGCCTAATCAAAGACTGAGGT |
| M00253 | V$CAP_01 | 0.937408 | 467 | (+) | NCANNNNN | TCAGCCTA |
| M00113 | V$CREB_02 | 0.789114 | 468 | (-) | NNGNTGACGYNN | CAGCCTAATCAA |
| M00244 | V$NGFIC_01 | 0.738296 | 468 | (-) | WTGCGTGGGYGG | CAGCCTAATCAA |
| M00246 | V$EGR2_01 | 0.757946 | 468 | (-) | NTGCGTRGGCGK | CAGCCTAATCAA |
| M00098 | V$PAX2_01 | 0.802315 | 469 | (-) | NNNNGTCANGNRTKANNNN | AGCCTAATCAAAGACTGAG |
| M00099 | V$S8_01 | 0.814918 | 469 | (-) | WNNANYYAATTANYNN | AGCCTAATCAAAGACT |
| M00137 | V$OCT1_03 | 0.855788 | 469 | (+) | NNNRTAATNANNN | AGCCTAATCAAAG |
| M00159 | V$CEBP_01 | 0.924149 | 470 | (-) | NNTKTGGWNANNN | GCCTAATCAAAGA |
| M00075 | V$GATA1_01 | 0.785785 | 471 | (-) | SNNGATNNNN | CCTAATCAAA |
| M00077 | V$GATA3_01 | 0.87284 | 471 | (-) | NNGATARNG | CCTAATCAA |
| M00254 | V$CAAT_01 | 0.796668 | 471 | (+) | NNNRRCCAATSA | CCTAATCAAAGA |
| M00160 | V$SRY_02 | 0.819143 | 472 | (+) | NWWAACAAWANN | CTAATCAAAGAC |
| M00162 | V$OCT1_06 | 0.814062 | 472 | (-) | CWNAWTKWSATRYN | CTAATCAAAGACTG |
| M00148 | V$SRY_01 | 0.962791 | 474 | (+) | AAACWAM | AATCAAA |
| M00238 | V$BARBIE_01 | 0.819448 | 474 | (+) | ATNNAAAGCNGRNGG | AATCAAAGACTGAGG |
| M00271 | V$AML1_01 | 0.910841 | 484 | (+) | TGTGGT | TGAGGT |

Notes: Estimating transcription factor bindability was by TFBIND.

Part II. TFBIND Results for the extra 48nt 5’UTR of CNR1

| TF Matrix ID | TF Label | Similarity | Position | Strandness | Consensus Sequence | Subsequence |
| --- | --- | --- | --- | --- | --- | --- |
| M00175 | V$AP4_Q5 | 0.799238 | 1 | (+) | NNCAGCTGNN | GTGAGCAGTG |
| M00175 | V$AP4_Q5 | 0.781284 | 1 | (-) | NNCAGCTGNN | GTGAGCAGTG |
| M00187 | V$USF_Q6 | 0.812133 | 3 | (-) | GYCACGTGNC | GAGCAGTGAT |
| M00237 | V$AHRARNT_02 | 0.719008 | 3 | (-) | GRGKATYGCGTGMSWNSCC | GAGCAGTGATGAGATCCTG |
| M00253 | V$CAP_01 | 0.875801 | 5 | (+) | NCANNNNN | GCAGTGAT |
| M00017 | V$ATF_01 | 0.762392 | 6 | (+) | CNSTGACGTNNNYC | CAGTGATGAGATCC |
| M00175 | V$AP4_Q5 | 0.785637 | 6 | (-) | NNCAGCTGNN | CAGTGATGAG |
| M00075 | V$GATA1_01 | 0.818855 | 7 | (+) | SNNGATNNNN | AGTGATGAGA |
| M00076 | V$GATA2_01 | 0.792061 | 7 | (+) | NNNGATRNNN | AGTGATGAGA |
| M00077 | V$GATA3_01 | 0.859548 | 8 | (+) | NNGATARNG | GTGATGAGA |
| M00113 | V$CREB_02 | 0.798054 | 8 | (+) | NNGNTGACGYNN | GTGATGAGATCC |
| M00221 | V$SREBP1_02 | 0.720902 | 8 | (-) | KATCACCCCAC | GTGATGAGATC |
| M00080 | V$EVI1_03 | 0.720782 | 9 | (+) | AGATAAGATAA | TGATGAGATCC |
| M00224 | V$STAT1_01 | 0.802239 | 10 | (+) | NNNSANTTCCGGGAANTGNSN | GATGAGATCCTGGAAGTGGAG |
| M00225 | V$STAT3_01 | 0.733092 | 10 | (+) | NGNNATTTCCSGGAARTGNNN | GATGAGATCCTGGAAGTGGAG |
| M00225 | V$STAT3_01 | 0.723534 | 10 | (-) | NGNNATTTCCSGGAARTGNNN | GATGAGATCCTGGAAGTGGAG |
| M00222 | V$TH1E47_01 | 0.806767 | 11 | (+) | NNNNGNRTCTGGMWTT | ATGAGATCCTGGAAGT |
| M00261 | V$OLF1_01 | 0.764422 | 11 | (+) | NNCNANTCCCYNGRGARNNKGN | ATGAGATCCTGGAAGTGGAGGT |
| M00075 | V$GATA1_01 | 0.82922 | 12 | (+) | SNNGATNNNN | TGAGATCCTG |
| M00075 | V$GATA1_01 | 0.788746 | 12 | (-) | SNNGATNNNN | TGAGATCCTG |
| M00076 | V$GATA2_01 | 0.862427 | 12 | (+) | NNNGATRNNN | TGAGATCCTG |
| M00076 | V$GATA2_01 | 0.84258 | 12 | (-) | NNNGATRNNN | TGAGATCCTG |
| M00032 | V$CETS1P54_01 | 0.874253 | 13 | (-) | NCMGGAWGYN | GAGATCCTGG |
| M00074 | V$CETS1P54_02 | 0.860602 | 16 | (+) | NNAMMGGAWRWNN | ATCCTGGAAGTGG |
| M00223 | V$STAT_01 | 0.856819 | 16 | (+) | TTCCCRKAA | ATCCTGGAA |
| M00032 | V$CETS1P54_01 | 0.905215 | 18 | (+) | NCMGGAWGYN | CCTGGAAGTG |
| M00108 | V$NRF2_01 | 0.844234 | 18 | (+) | ACCGGAAGNS | CCTGGAAGTG |
| M00255 | V$GC_01 | 0.802793 | 20 | (+) | NRGGGGCGGGGCNK | TGGAAGTGGAGGTG |
| M00183 | V$MYB_Q6 | 0.846058 | 21 | (-) | NNNAACKGNC | GGAAGTGGAG |
| M00196 | V$SP1_Q6 | 0.753551 | 23 | (+) | NGGGGGCGGGGYN | AAGTGGAGGTGGC |
| M00243 | V$EGR1_01 | 0.781281 | 23 | (+) | WTGCGTGGGCGK | AAGTGGAGGTGG |
| M00244 | V$NGFIC_01 | 0.797845 | 23 | (+) | WTGCGTGGGYGG | AAGTGGAGGTGG |
| M00255 | V$GC_01 | 0.781725 | 23 | (+) | NRGGGGCGGGGCNK | AAGTGGAGGTGGCA |
| M00002 | V$E47_01 | 0.817999 | 24 | (+) | NSNGCAGGTGKNCNN | AGTGGAGGTGGCAGA |
| M00073 | V$DELTAEF1_01 | 0.819038 | 26 | (-) | NNNCACCTNAN | TGGAGGTGGCA |
| M00255 | V$GC_01 | 0.797893 | 29 | (+) | NRGGGGCGGGGCNK | AGGTGGCAGAATGT |
| M00007 | V$ELK1_01 | 0.780227 | 31 | (+) | NNNACMGGAAGTNCNN | GTGGCAGAATGTGGGA |
| M00008 | V$SP1_01 | 0.824179 | 31 | (+) | GRGGCRGGGW | GTGGCAGAAT |
| M00260 | V$HLF_01 | 0.826171 | 31 | (-) | RTTACRYAAT | GTGGCAGAAT |
| M00032 | V$CETS1P54_01 | 0.818305 | 34 | (+) | NCMGGAWGYN | GCAGAATGTG |
| M00253 | V$CAP_01 | 0.871858 | 34 | (+) | NCANNNNN | GCAGAATG |
| M00055 | V$NMYC_01 | 0.745438 | 35 | (-) | NNNCACGTGNNN | CAGAATGTGGGA |
| M00187 | V$USF_Q6 | 0.800951 | 36 | (-) | GYCACGTGNC | AGAATGTGGG |
| M00141 | V$LYF1_01 | 0.864298 | 40 | (+) | TTTGGGAGR | TGTGGGAAG |

Notes: Estimating transcription factor bindability was by TFBIND. Green color highligghted 3 kinds of new binding sites
