# Supplementary figures and images for: Cannabinoid receptor CNR1 expression and DNA methylation in human prefrontal cortex, hippocampus and caudate in brain development and schizophrenia
Source: Transl Psychiatry. 2020 May 19;10:158. doi: 10.1038/s41398-020-0832-8 (PMC7237456; doi:10.1038/s41398-020-0832-8)

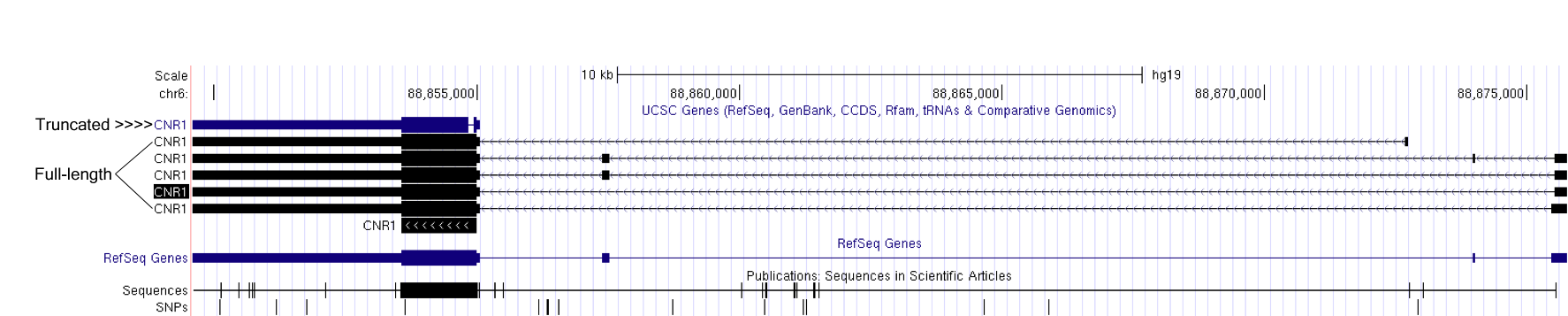

Supplement: Supplementary file 6 — Supplementary Figure 1 [file 41398_2020_832_MOESM6_ESM.tif]

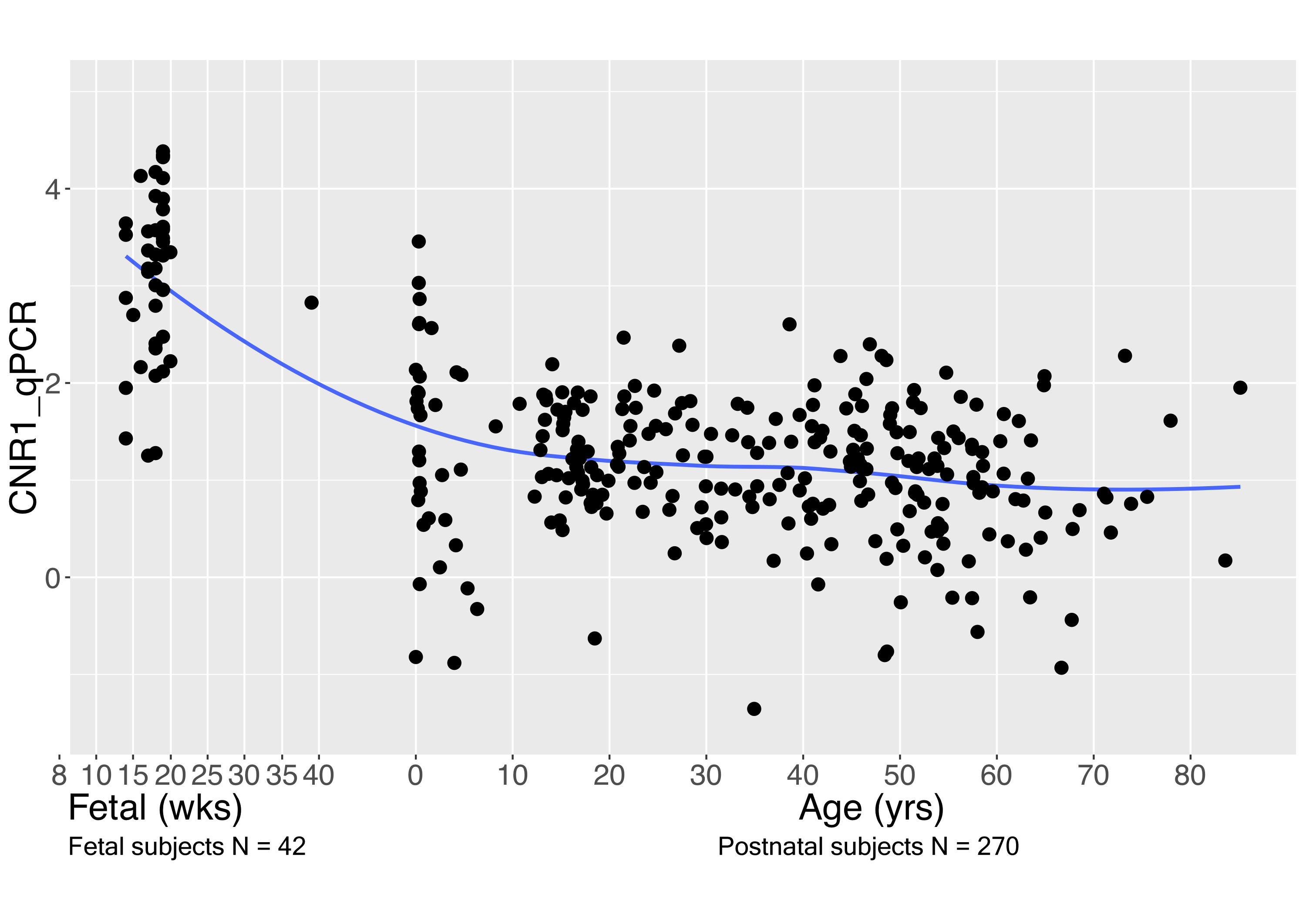

Supplement: Supplementary file 7 — Supplementary Figure 2 [file 41398_2020_832_MOESM7_ESM.tif]

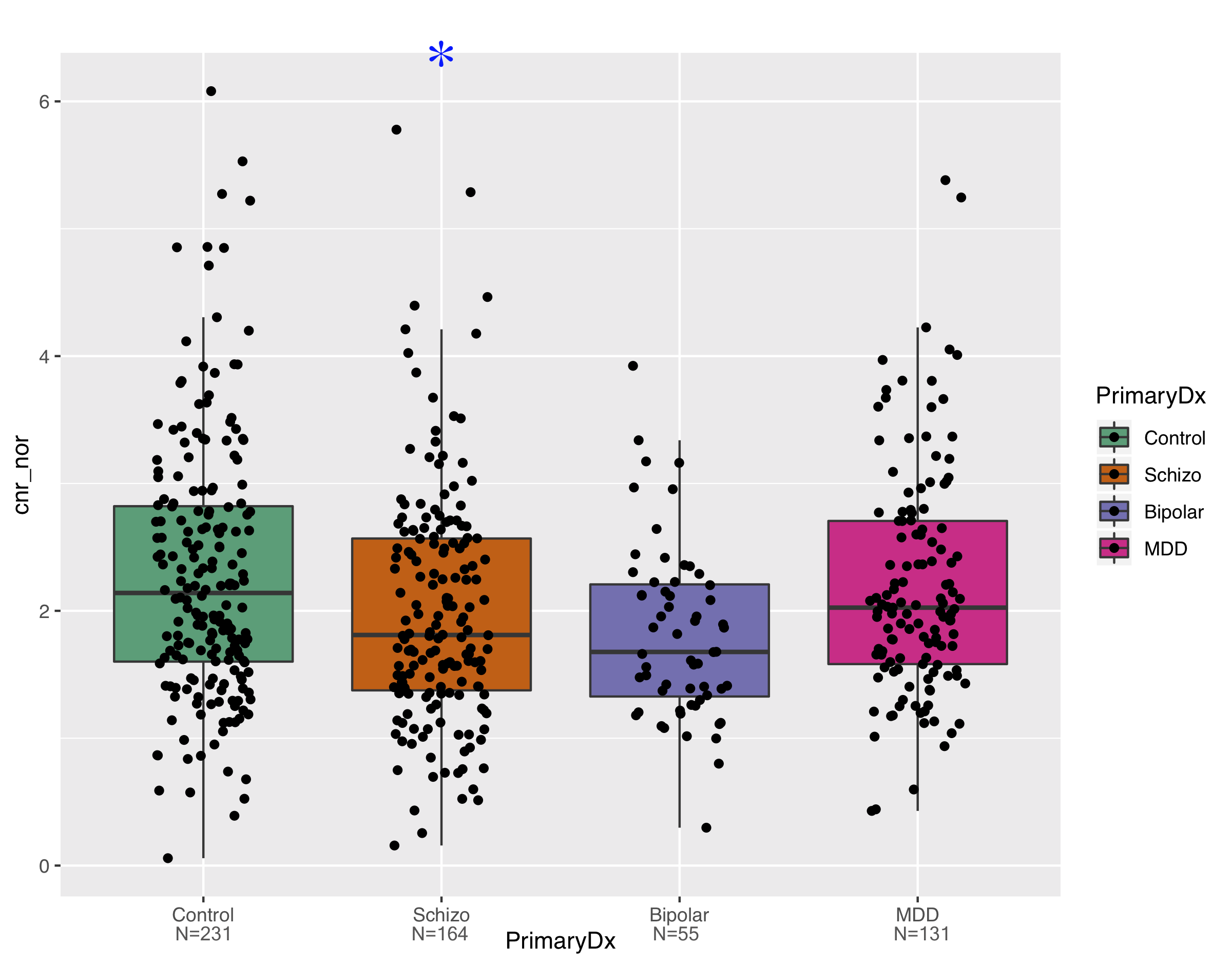

Supplement: Supplementary file 8 — Supplementary Figure 3 [file 41398_2020_832_MOESM8_ESM.tif]

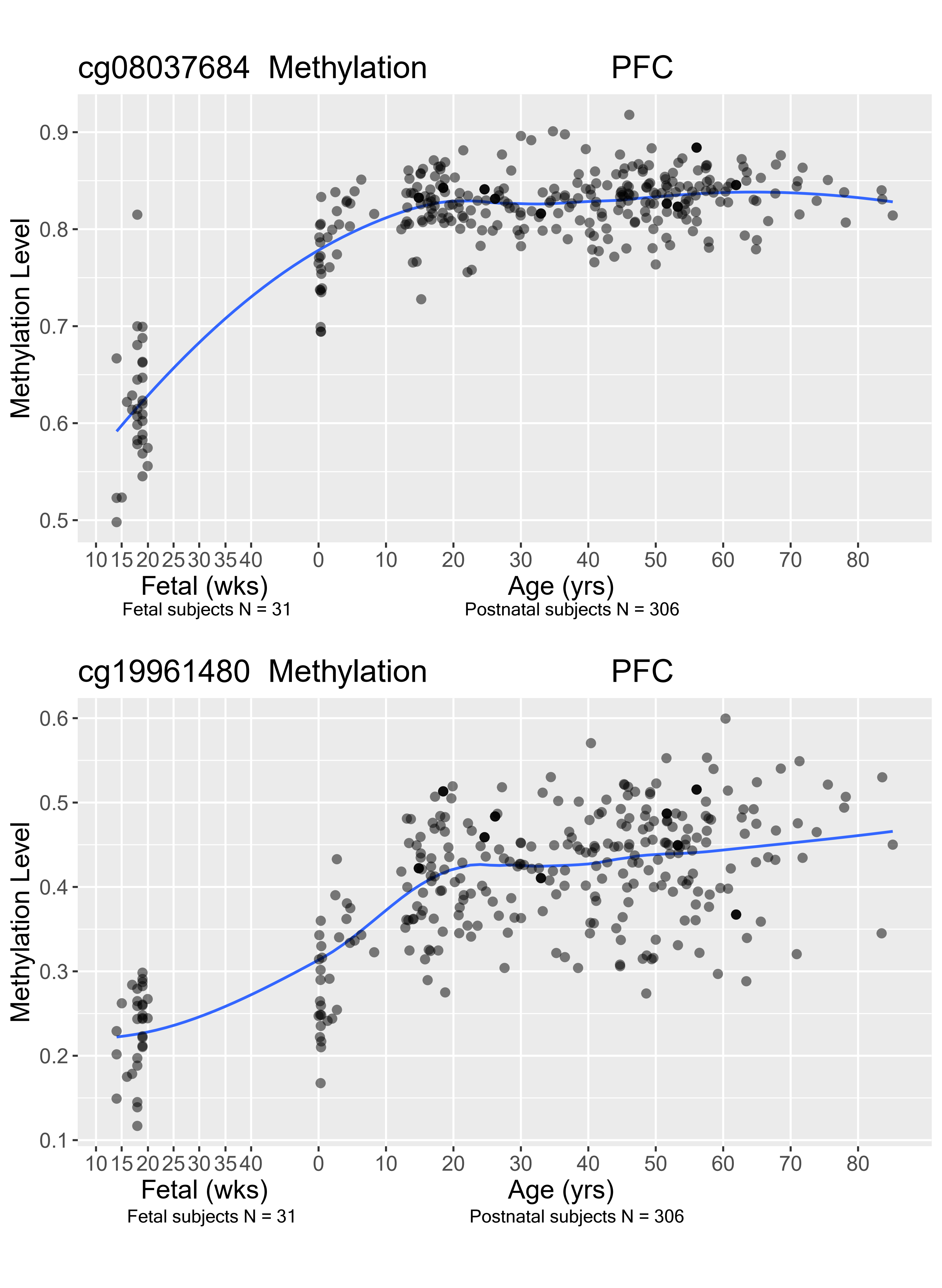

Supplement: Supplementary file 9 — Supplementary Figure 4 [file 41398_2020_832_MOESM9_ESM.tif]

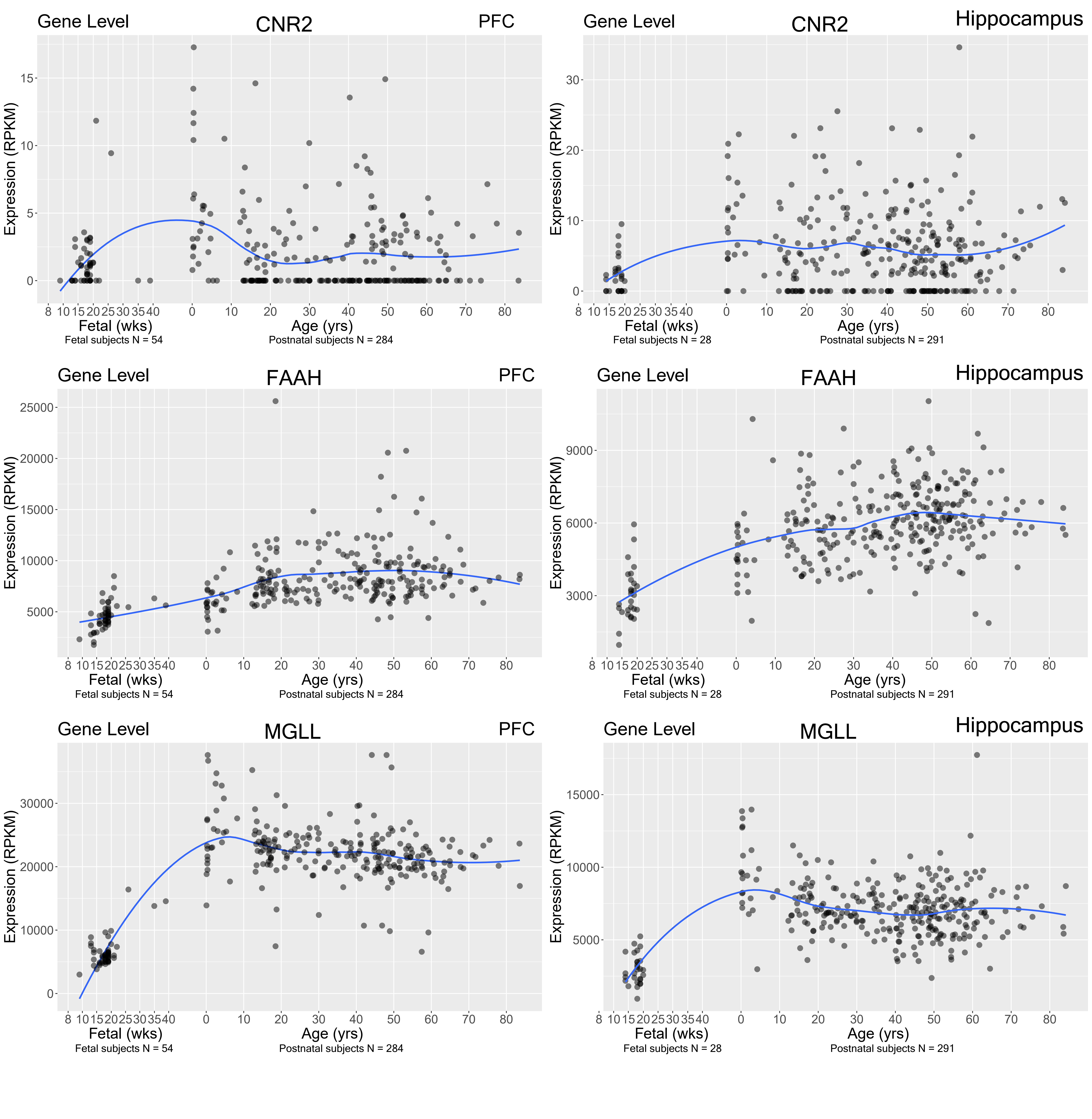

Supplement: Supplementary file 10 — Supplementary Figure 5 [file 41398_2020_832_MOESM10_ESM.tif]
